# Genetic Anchoring of Stromal Cellular Programs Relevant to Ligamentum Flavum Remodeling in Lumbar Spinal Stenosis

**DOI:** 10.3390/genes17101281

**Published:** 2026-10-14

**Authors:** Shihe Chen, Qinglin Bai, Yufei Hua, Dong Chen, Xin Jiang

**Affiliations:** 1 Peking University China-Japan Friendship School of Clinical Medicine, Beijing 100029, China; shihechen2002@163.com; 2 China-Japan Friendship Clinical Medical College, Beijing University of Chinese Medicine, Beijing 100029, China; 20250931881@bucm.edu.cn; 3 China-Japan Friendship Hospital, Chinese Academy of Medical Sciences & Peking Union Medical College, Beijing 100029, China; hyf07052002@163.com; 4 Department of Orthopaedics, China-Japan Friendship Hospital, No. 2 Yinghua East Street, Chaoyang District, Beijing 100029, China; gdcd2006@sina.com

**Keywords:** lumbar spinal stenosis, ligamentum flavum, genetics, single-cell RNA sequencing, *CRLF1*

## Abstract

Background/Objectives: Lumbar spinal stenosis (LSS) has a substantial inherited component, but the cellular contexts in which genetic susceptibility acts remain incompletely resolved. We integrated large-scale human genetics with a donor-resolved spinal-ligament single-cell resource and an external ligamentum flavum (LF) expression dataset to localize inherited susceptibility and prioritize regulatory candidates. Methods: We reconstructed a nine-donor spinal-ligament single-cell resource and derived donor-aware Broad Stromal and degenerative-LSS-associated Stromal transcriptional programs. These programs were evaluated using stratified LD score regression (S-LDSC) against an LSS genome-wide association dataset of 40,303 cases and 741,468 controls, followed by gene-based association, fine-mapping, target-aware molecular colocalization, donor-robustness analyses, and external LF tissue directional sensitivity. Results: The Broad Stromal annotation was the strongest and only multiple-testing-significant positive annotation in the primary prespecified one-at-a-time S-LDSC analysis (coefficient = 6.48 × 10^−8^, *p* = 0.0031, BH-FDR = 0.0245), whereas the disease-associated Stromal program was not significantly enriched. Donor-aware Stromal pseudobulk analysis identified 106 differentially expressed genes at FDR < 0.05, with high leave-one-donor-out directional stability. Cross-layer integration prioritized six genes. *CRLF1* showed the most complete candidate-specific regulatory convergence, including an eight-variant credible set and GTEx skeletal-muscle *CRLF1* colocalization at rs2074177 (PP.H4 = 0.974), while *MMP14* showed stronger disease-associated differential expression and a narrower two-variant credible set. Conclusions: Inherited LSS susceptibility preferentially localizes to a broad spinal-ligament Stromal cellular/regulatory context rather than establishing genetic mediation of the observed disease-associated transcriptional state. *CRLF1* and *MMP14* represent complementary candidates for tissue-specific functional validation.

## 1. Introduction

Lumbar spinal stenosis (LSS) is a common and disabling disorder of older adults, characterized by narrowing of the lumbar spinal canal or neural foramina and frequently presenting with back or leg pain, neurogenic claudication, and impaired mobility [1,2]. Its clinical burden is substantial, with more than 100 million people estimated to be affected worldwide and large numbers of patients ultimately undergoing decompressive surgery [1,3]. Degenerative LSS is anatomically heterogeneous and reflects changes across multiple spinal structures, including the intervertebral disks, facet joints, vertebrae, and ligamentum flavum (LF) [1,2,4]. Among these, LF thickening and fibrosis can directly reduce canal reserve and contribute to neural compression [4,5]. Histopathologically, hypertrophic LF is characterized by loss of elastic architecture, increased collagen deposition, and extracellular-matrix remodeling, linking local tissue degeneration to the clinical stenotic phenotype [4,5].

Accumulating molecular and single-cell studies have begun to resolve the cellular programs underlying LF degeneration [4,6]. Experimental work has implicated mechanical stress, inflammatory and profibrotic signaling, and fibroblast-like remodeling in LF hypertrophy [4,7,8]. *CRLF1*, for example, has been shown to be upregulated in hypertrophic LF and to participate in TGF-β1/SMAD3- and ERK-related fibrotic responses in vitro and in vivo [9]. More recently, single-cell RNA sequencing has revealed marked heterogeneity within degenerative spinal ligaments [6]. Tang et al. identified stromal and immune subpopulations associated with matrix degradation, chondrogenic/calcific remodeling, and inflammatory activation, including CRTAC1-positive chondrocyte-like cells and SPP1-positive macrophages [6]. Together, these studies define a detailed disease-state landscape of LF biology and motivate testing which cellular programs are preferentially connected to inherited susceptibility to LSS [4,6].

In parallel, human genetic studies have substantially expanded the genomic architecture of LSS [3,10]. Earlier transcriptome-wide association analyses linked genetically predicted expression in skeletal muscle and blood to candidate LSS genes [10]. More recently, a large genome-wide meta-analysis comprising more than 40,000 LSS cases and over 740,000 controls identified 88 associated loci, including 73 previously unreported loci, and reinforced a substantial inherited component to LSS risk [3]. This expanded locus map creates an opportunity to resolve the tissue and cellular contexts in which susceptibility variants act and to prioritize biologically relevant effector genes [11]. A key unresolved question is therefore whether the expanding genetic architecture of LSS converges on specific spinal-ligament cellular programs relevant to LF remodeling and whether such localization can help prioritize biologically coherent genes within associated loci [3,11].

The present study therefore does not aim to rediscover additional stromal or immune populations. Instead, we use a donor-resolved spinal-ligament single-cell resource to define broad cellular programs and ask a complementary genetics-first question: where does inherited LSS susceptibility preferentially localize? We then integrate program-level genetic localization with donor-aware degenerative-LSS-associated transcription, gene-based association, fine-mapping, target-aware molecular colocalization, and external LF tissue directional sensitivity. By treating these evidence layers as complementary but non-equivalent, the framework extends prior transcriptomic descriptions from disease-state characterization toward genetic contextualization of LSS susceptibility and locus-level candidate prioritization.

## 2. Materials and Methods

### 2.1. Study Design and Data Sources

#### 2.1.1. Overall Analytical Framework

This study used an integrative, genetics-first design to determine whether inherited susceptibility to lumbar spinal stenosis (LSS) preferentially localizes to disease-relevant cellular programs defined from a spinal-ligament single-cell resource [12]. The analysis was organized into five linked but analytically distinct layers: reconstruction of the spinal-ligament single-cell resource, donor-aware definition of broad cellular and degenerative-LSS-associated transcriptional programs, genetic localization of these programs using stratified LD score regression (S-LDSC), gene- and locus-level evidence integration, and external LF tissue directional-sensitivity analysis [13,14]. Evidence from each layer was retained as a separate category; in particular, Broad Stromal identity, disease-associated Stromal transcription, and locus-level molecular colocalization were not treated as a demonstrated causal or mediation chain. All analyses used de-identified, publicly available data; no new participants were recruited and no new biological specimens were collected.

#### 2.1.2. LSS Genome-Wide Association Data

The primary genetic dataset was the overall LSS genome-wide association study reported by Salo et al., comprising 40,303 LSS cases and 741,468 controls [3]. The downloaded summary statistics contained 15,144,278 variants and were provided in GRCh38 coordinates with chromosome, position, reference/effect alleles, effect-allele frequency, effect size, standard error, *p* value, and rsID fields [3]. The overall LSS phenotype was used as the primary genetic outcome throughout the present study. For S-LDSC, GWAS variants were harmonized to the LD reference through rsID and allele compatibility, providing the coordinate linkage required for S-LDSC without whole-file coordinate conversion.

#### 2.1.3. Spinal-Ligament Single-Cell and LF Transcriptomic Resources

Single-cell analyses used the publicly available GEO dataset GSE271018. The source study describes the nine samples conservatively as spinal-ligament/ligament-tissue samples rather than establishing that every library was derived exclusively from ligamentum flavum [6]. The source cohort comprised six degenerative-LSS donors (ages 49, 51, 74, 51, 57, and 60 years; four women and two men) and three traumatic controls (ages 57, 51, and 56 years; one woman and two men), with lumbar sampling levels spanning mainly L3–L5/L4–L5 in the degenerative group and L1–L3 in controls. Donor-level metadata available for the present analysis included age, sex, sample group, and lumbar sampling level; stenosis severity and sufficiently detailed trauma-related clinical variables were not available. The original study sorted CD45-positive and CD45-negative cells before sequencing and subsequently mixed the two fractions at a fixed ratio; therefore, the reconstructed resource was used for cellular-state definition and cell proportions were excluded from native-abundance analyses [6]. The downloaded donor-level objects contained 51,686 cells before uniform re-QC, of which 43,044 were retained (Supplementary Table S1). External tissue directional-sensitivity analysis used GSE113212 (GPL17077), which contained four young/non-hypertrophic LF samples and four elderly/hypertrophic LF samples. Because age and LF hypertrophy status are completely aligned in GSE113212, age matching or reliable separation of age from hypertrophy is not possible within this eight-sample design.

### 2.2. Single-Cell Atlas Reconstruction and Major Cell-Type Annotation

#### 2.2.1. Uniform Quality Control and Atlas Reconstruction

The nine donor-level Seurat objects from GSE271018 were reprocessed from raw RNA counts using a uniform pipeline. When doublet-classification metadata were available, only singlets were retained. Cells were required to have mitochondrial transcript percentage ≤ 10%, total RNA counts ≥ 500, and detected RNA features ≥ 200; no upper nCount_RNA threshold was imposed. After quality control, 43,044 cells and 25,811 features were retained. The donor-level objects were merged, normalized using LogNormalize, and the 2000 most variable features were selected. Expression values were scaled and principal-component analysis was performed using 50 principal components [15]. Harmony was then applied using donor identity as the integration variable [16]. A nearest-neighbor graph was constructed using the first 30 Harmony dimensions, clustering resolution was scanned from 0.2 to 1.2, and a working resolution yielding 19 reconstructed clusters was selected for marker-based biological annotation. Uniform Manifold Approximation and Projection (UMAP) was used for low-dimensional visualization [15].

#### 2.2.2. Broad Cell-Type Annotation

Cluster identities were assigned by manual review of canonical lineage markers. Major atlas populations included Stromal, Myeloid, Neutrophil, proliferating neutrophil, T-cell, B-cell, Endothelial, Pericyte, and hematopoietic stem/progenitor populations. Representative marker sets included *COL1A1*, *DCN*, and *LUM* for Stromal cells; *LST1*, *TYROBP*, and *FCER1G* for Myeloid cells; *FCGR3B*, *S100A8*, and *S100A9* for Neutrophils; *MKI67*, *TOP2A*, and *STMN1* for proliferating neutrophils; *CD3D*, *CD3E*, and *TRBC1* for T cells; *CD79A*, *MS4A1*, and *CD74* for B cells; *PECAM1*, *VWF*, and *EMCN* for Endothelial cells; *RGS5*, *CSPG4*, and *PDGFRB* for Pericytes; and *CD34*, *KIT*, and *GATA2* for hematopoietic stem/progenitor cells. Fine-grained Stromal and macrophage substates were explored during reconstruction; broader marker-supported cellular compartments showed the greatest reproducibility and therefore formed the primary analytical framework [6].

### 2.3. Donor-Aware Transcriptional Program Construction

#### 2.3.1. Broad Cell-Type Identity Programs

Broad cell-type programs used for genetic localization were derived from raw counts with donor, rather than individual cell, treated as the biological replicate [13]. Reconstructed clusters were grouped into eight prespecified broad lineages: Stromal, Myeloid, T/NK, B lineage, Neutrophil, Endothelial, Pericyte, and conventional dendritic cell (cDC). Erythroid and HSC-like clusters were excluded from the primary broad-program analysis because they did not meet the predefined lineage eligibility framework. For each donor–lineage combination, at least 20 cells were required for pseudobulk aggregation, and each lineage required at least three eligible donors. Raw counts were summed across cells within each donor–lineage pair. Genes were filtered with edgeR filterByExpr, library sizes were normalized by the trimmed mean of M values (TMM), and a donor-blocked quasi-likelihood model of the form ~0 + broad_celltype + donor was fitted [13]. For each target lineage k, its model coefficient was contrasted against the equally weighted mean of the other K-1 eligible lineage coefficients: $C_{k}=\beta_{k}-\frac{1}{K-1}\;\sum_{l\neq k}\beta_{l}\;$. For each gene g and lineage k, the two-sided edgeR *p* value was converted to a signed normal-equivalent statistic using $p_{safe}=max(p,10^{-300})$ and $z_{g,k}=sign(logFC_{g,k})\Phi^{-1}(1-\frac{p_{safe}}{2})$. This transformation re-expresses the edgeR evidence on a signed standard-normal scale and does not assume that the log fold change itself is normally distributed.

#### 2.3.2. Disease-Associated Stromal Transcription

Disease-associated transcription was assessed separately from Broad Stromal identity. Raw counts were aggregated to donor-by-compartment pseudobulk profiles for the Stromal and Myeloid compartments, requiring at least 20 cells from a donor for inclusion in the corresponding compartment [13]. Differential expression was estimated with edgeR quasi-likelihood models for degenerative LSS versus traumatic control [13]. For the Stromal compartment, eligibility filtering yielded eight donors (five degenerative LSS and three traumatic controls). The final frozen analysis was restricted to autosomal genes and used a sex-adjusted model (~ sex + group), with donor as the biological replicate. Because only one traumatic control was female, sex adjustment reduces but cannot eliminate residual sex-group confounding. Donor influence was assessed by leave-one-donor-out (LODO) refitting, and a male-only analysis of two LSS and two traumatic-control donors was retained strictly as a directional-sensitivity analysis rather than independent replication. A sex-plus-age model was also evaluated as a sensitivity analysis; effect rankings and directions were highly concordant with the primary model, but the additional covariate reduced residual degrees of freedom and statistical power. Benjamini–Hochberg false-discovery rate (FDR) correction was applied across tested genes within each compartment (Supplementary Table S2).

### 2.4. Genetic Anchoring of Spinal-Ligament-Derived Cellular Programs

#### 2.4.1. Program Weighting and SNP-to-Gene Mapping

Continuous broad cell-type programs were transformed to non-negative 0–1 gene weights following the sc-linker/GSSG processing framework [12]. For each signed score z, $z_{plus}=max(z,0)$, $p^{*}=2\Phi (-|z_{plus}|)$, $X=-2ln(p^{*}+10^{-8})$, $w=\frac{X\;-\;X_{min}}{X\;-\;X_{max}}$, constrained to [0, 1] within each program. Negative values were set to zero because they represent depletion rather than enrichment in the target lineage. Non-finite signed scores were prospectively mapped to zero before transformation; continuous ties were retained without jitter. The 1 × 10^−8^ offset caps the pre-scaling X statistic at approximately 36.84, so extreme upper-tail signals can share identical or near-identical weights; these weights are annotation strengths, not causal probabilities [12]. Binary Top500, Top1000, and Top1500 sensitivity sets were ranked among positively enriched genes by ascending edgeR *p* value, decreasing logFC, and gene symbol as a deterministic final tie-breaker. The primary SNP-to-gene mapping used ABC_Road_ALL, defined as the union of Activity-By-Contact (ABC) enhancer-gene predictions and Roadmap enhancer-gene links across available epigenomic contexts [11,12]. This was used as a broad regulatory linking scaffold, not as an LF- or spinal-ligament-specific enhancer map; cellular specificity arose from the donor-aware gene-program weights. Because no experimentally defined spinal-ligament/LF enhancer-gene map was available, we did not select an unvalidated surrogate tissue. A conventional 100 kb gene-window strategy was generated independently as an orthogonal mapping sensitivity analysis [12]. For overlapping genomic intervals, the maximum linked gene-program weight was retained.

#### 2.4.2. Partitioned S-LDSC Analysis

Partitioned S-LDSC was performed using the baselineLD v2.2 model, 1000 Genomes Project Phase 3 European LD reference data, and HapMap3 regression SNPs with the MHC region excluded from the regression weights [17]. The primary analysis evaluated the eight continuous broad cell-type annotations using ABC_Road_ALL, one custom annotation at a time conditional on baselineLD v2.2 [12,17]. Because the custom annotations were continuous, the conditioned annotation coefficient, its standard error, Z statistic, and two-sided *p* value were treated as the primary S-LDSC statistics [17]. Benjamini–Hochberg correction was applied across the eight prespecified broad cellular programs. The Stromal result was challenged using an alternative continuous 100 kb mapping and nested Top500/Top1000/Top1500 binary Stromal annotations using 100 kb mapping [12]. We additionally quantified Pearson correlations among the eight custom-program LD-score predictors across common SNPs, fitted seven pairwise conditional models containing Broad Stromal plus one companion annotation together with baselineLD v2.2, and used model covariance matrices for exploratory coefficient-difference tests. An exploratory joint model containing all eight broad programs with baselineLD v2.2 was also fitted to assess uncertainty under shared annotation structure; these correlation and conditional analyses are summarized in Supplementary Figure S1. Separately, the disease-associated Stromal and Myeloid programs were tested under both ABC_Road_ALL and 100 kb mapping; these secondary analyses were not interpreted as tests of mediation (Supplementary Figure S3).

### 2.5. Gene-Level Genetic Evidence Integration and Candidate Prioritization

#### 2.5.1. Published Locus, Gene-Test, Fine-Mapping, and Colocalization Evidence

Gene-level genetic evidence was imported from the Supplementary Datasets accompanying the Salo et al. LSS GWAS [3]. Specifically, Supplementary Data 2 provided reported LSS loci, lead variants, and candidate genes; Supplementary Data 5 provided fine-mapping credible-set information; Supplementary Data 10 contained GCTA-fastBAT gene-based association results; Supplementary Data 11 contained MAGMA gene-based association results; and Supplementary Data 12 contained colocalization results [3]. Candidate-gene and gene-test evidence were linked by exact gene symbol. Fine-mapping and colocalization evidence were linked to the candidate table only by exact LSS lead-rsID match. The canonical Supplementary Data 5 table used in this study reports CHR:POS, OA, EA, rsID, credible-set size, and variant type but does not contain a per-variant posterior-probability column; therefore, no variant-level posterior probability was inferred or imputed. Rows without an exact-rsID match were classified as unavailable exact linkage in the present audit.

#### 2.5.2. Transparent Evidence Strata and Shortlist Definition

A small manuscript shortlist was frozen using transparent, prespecified evidence strata. Stratum A required a Salo candidate gene, significant fastBAT support, significant MAGMA support, and positive Broad Stromal weight. Stratum B required a Salo candidate gene, at least one significant gene-based test (fastBAT or MAGMA), positive Broad Stromal weight, and a frozen Stromal disease tier meeting FDR < 0.10. Stratum C required a Salo candidate gene, at least one significant gene-based test, positive Broad Stromal weight, and nominal but directionally retained Stromal disease transcription. The union of these three strata yielded the six-gene shortlist *BMP6*, *CRLF1*, *JAZF1*, *MMP14*, *OTUD7B*, and *SOX5*. Previously reported LF remodeling genes were maintained as a complementary mechanistic/contextual panel [6].

#### 2.5.3. Within-Program Cellular Localization Audit

For each shortlisted gene, the frozen continuous weight was recovered across all eight broad cellular programs and converted to a within-program percentile [12]. These percentiles summarized the relative position of each gene within each program-specific ranking. Gene-level genetics, fine-mapping, molecular colocalization, and disease-associated transcription were displayed as complementary evidence layers to support cross-layer candidate prioritization.

### 2.6. Molecular Colocalization Adjudication and Regional CRLF1 Analysis

#### 2.6.1. Target-Aware Colocalization Adjudication

Exact-rsID colocalization rows from Salo Supplementary Data 12 were adjudicated according to the biological identity of the paired trait [3]. A colocalization result was classified as candidate-gene-specific molecular evidence only when the LSS lead rsID matched exactly, the paired dataset represented a molecular QTL, and the reported molecular target matched the candidate gene itself. Molecular-QTL colocalizations implicating another gene at the same locus were retained as alternative-target evidence rather than reassigned to the candidate gene. Colocalizations with clinical phenotypes were retained as phenotype-GWAS locus context only. Under this target-aware audit, *CRLF1* was the only shortlisted gene with candidate-gene-specific molecular colocalization across multiple molecular-QTL resources; alternative molecular targets were observed at several other loci [3].

#### 2.6.2. Regional *CRLF1* Locus Visualization

The *CRLF1* locus was examined at regional resolution around rs2074177 (chr19:18,616,479, GRCh38) [3]. LSS GWAS variants within ±500 kb of the lead variant were extracted from the overall LSS summary statistics [3]. Variant-level *CRLF1* cis-eQTL data were obtained from the eQTL Catalogue GTEx skeletal-muscle gene-expression dataset (study QTS000015, dataset QTD000281; *CRLF1* ENSG00000006016) over the same interval [14]. After schema and rsID audit, 4817 GWAS variants and 3314 *CRLF1* cis-eQTL variants were available, with 2651 overlapping rsIDs. Across all overlaps, the GWAS effect allele matched the eQTL ALT/effect allele and the GWAS other allele matched the eQTL reference allele. At rs2074177, the LSS GWAS reported OA = A, EA = G, beta = −0.0649, whereas the eQTL reported REF = A, ALT = G, beta = +0.397042; thus, G is protective and associated with higher *CRLF1* expression, while the LSS risk-increasing A allele corresponds to lower *CRLF1* expression in GTEx skeletal muscle. The variant is non-palindromic (A/G). The displayed PP.H4 = 0.974 corresponds to the audited candidate-gene-specific GTEx skeletal-muscle *CRLF1* gene-expression colocalization [3,14]. PP.H4 was interpreted as posterior support for a shared association signal under the colocalization model, not as the causal probability of rs2074177 and not as proof of mediation through *CRLF1* (Supplementary Figure S5).

### 2.7. External LF Tissue Sensitivity Analysis

#### 2.7.1. Resource Audit and Gene-Level Preprocessing

GSE113212 was retrieved as a GEO series-matrix ExpressionSet on platform GPL17077. Sample titles defined four Young_nonhypertrophic and four Elderly_hypertrophic LF samples. The platform annotation was audited before analysis. Probe-to-gene mapping used the GENE_SYMBOL annotation; probes mapping ambiguously to multiple genes were excluded, and the median expression across eligible probes was used when multiple unique probes mapped to the same gene. The analysis was restricted a priori to the frozen six-gene shortlist together with five previously examined LF remodeling genes (*SPP1*, *ATF3*, *CRTAC1*, *MGP*, and *CLU*); the external dataset was analyzed as a prespecified frozen-panel expression resource [6]. Because the available design contains no young/hypertrophic or elderly/non-hypertrophic samples, age and hypertrophy status are completely aligned. Age matching is therefore infeasible, and an age-adjusted model cannot robustly separate aging from hypertrophy in this eight-sample dataset.

#### 2.7.2. Frozen-Panel Differential Expression and Directional Concordance

Differential expression in GSE113212 was tested with limma using the contrast Elderly_hypertrophic minus Young_nonhypertrophic. Benjamini–Hochberg correction was applied only across the evaluable genes in the prespecified frozen panel. Directional concordance was then assessed by comparing the sign of the external log fold change with the corresponding internal donor-aware Stromal disease result when an internal direction was available. FDR categories and directional concordance were used to summarize cross-dataset consistency. The external analysis was designated a tissue-level sensitivity analysis, reflecting the complete alignment of age and LF hypertrophy in this dataset.

### 2.8. Statistical Analysis, Evidence Hierarchy, and Reproducibility

#### 2.8.1. Multiple Testing and Evidence Interpretation

All reported *p* values were two-sided unless otherwise specified. Multiple testing was controlled with the Benjamini–Hochberg procedure within each prespecified analysis family: eight broad cellular programs for the primary S-LDSC analysis, three nested Top-K Stromal sensitivity annotations, genome-wide tested genes within the donor-aware disease-expression analysis, and the frozen evaluable gene panel in GSE113212. Evidence categories were kept conceptually distinct throughout the study. S-LDSC was interpreted as cell-program-level genetic localization; disease-associated transcription as state-dependent expression evidence; fastBAT and MAGMA as gene-based association evidence; fine-mapping as variant/locus narrowing; molecular colocalization as compatibility with a shared association signal; and external bulk LF expression as directional sensitivity [3,12,13,17]. Together, these layers provided a stepwise evidence hierarchy from cell-program localization to locus-level candidate prioritization.

#### 2.8.2. Software and Reproducibility

Single-cell processing was performed in R (v4.6.0) using Seurat (v5.5.1) and Harmony (v2.0.5) [15,16]. Pseudobulk modeling used edgeR (v4.10.1), external microarray analysis used limma (v3.68.4), and data processing and visualization used standard R packages including data.table (v1.18.6.1; https://r-datatable.com (accessed on 11 October 2026)) and ggplot2 (v4.0.3) [13]. S-LDSC was run with LDSC v3.0.1 under Python 3.9.23 using baselineLD v2.2 and 1000 Genomes European reference resources [12,17]. The annotation-correlation audit used Python 3.13.13 with pandas 3.0.3 and NumPy 2.5.1. RStudio (v2026.6.0.242; https://posit.co/products/open-source/rstudio/ (accessed on 11 October 2026)) was used as the R development environment. Key analytical stages were accompanied by explicit input audits, frozen intermediate tables, and gate checks before downstream use. A portable R helper reproducing the frozen broad-program signed-score, weight, and Top-K transformations is included in the revision materials. The versioned public analysis repository, including program-score manifests, S-LDSC commands and key outputs, donor-robustness summaries, candidate-evidence tables, README, and environment information, is available at https://github.com/csh-art/LSS-stromal-genetics (accessed on 11 October 2026) and archived on Zenodo (version v1.0.0-revision; DOI: 10.5281/zenodo.23213035).

## 3. Results

### 3.1. Single-Cell Reconstruction Defines the Major Cellular Landscape of the Spinal-Ligament Resource

**Figure 1:**
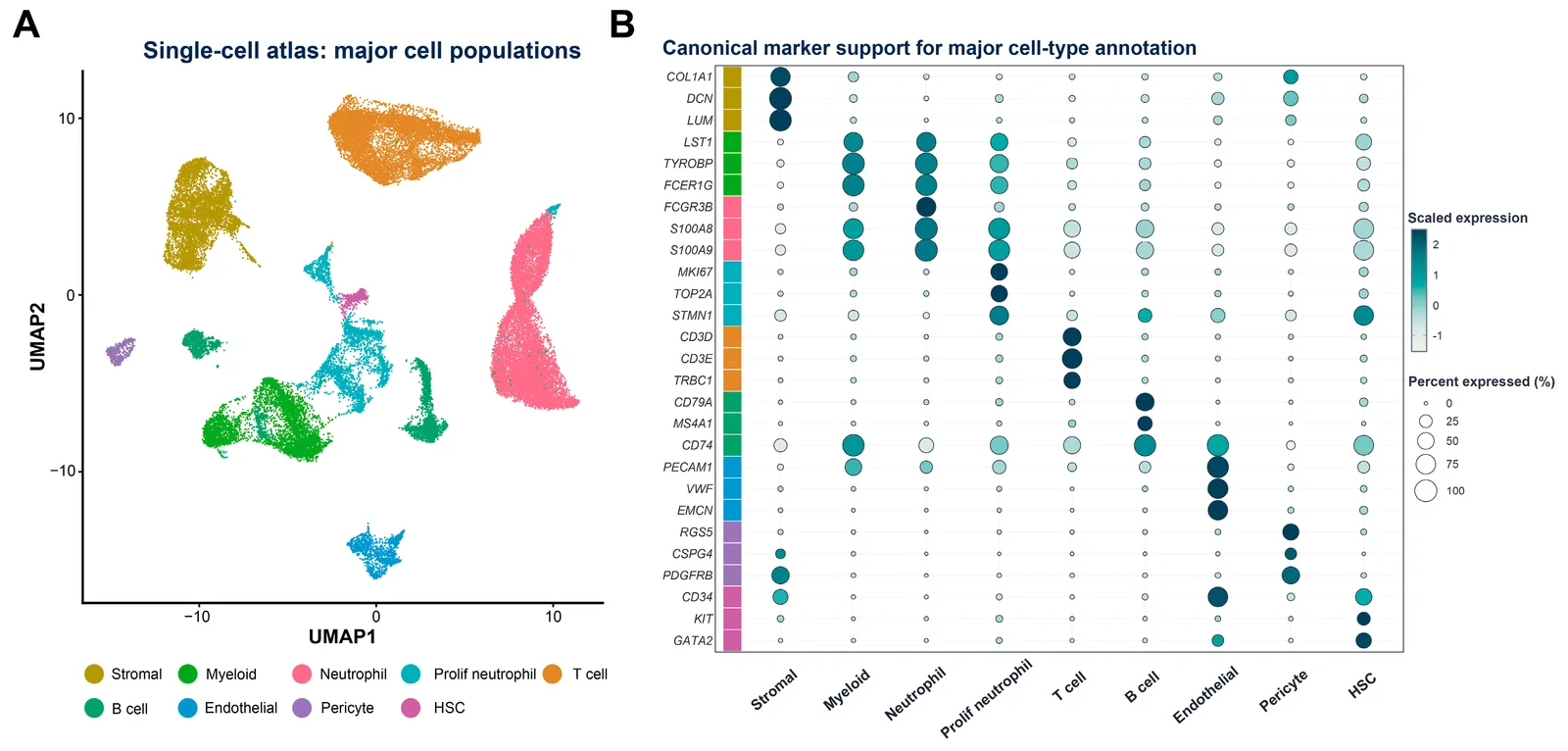
Single-cell reconstruction and major cell-type annotation of the GSE271018 spinal-ligament/ligament-tissue resource. (**A**) UMAP representation of the reconstructed nine-donor resource showing major cellular populations. (**B**) Canonical-marker dot plot supporting broad cell-type annotations; dot size represents the percentage of cells expressing each marker and color represents scaled expression. The source material does not establish that every donor library was exclusively derived from ligamentum flavum. In panel (**A**), different colors distinguish the annotated major cell populations.

We reconstructed the cellular landscape represented in the nine-donor GSE271018 spinal-ligament/ligament-tissue single-cell resource [6]. After unified quality control, dimensionality reduction, batch correction, and clustering, 43,044 cells were retained for downstream analysis. Major cell populations were annotated on the basis of canonical marker expression and included Stromal, Myeloid, Neutrophil, proliferating neutrophil, T-cell, B-cell, Endothelial, Pericyte, and hematopoietic stem/progenitor populations (Figure 1A). Because donor-level source information does not establish exclusive LF origin for every library, all GSE271018-derived cell-program analyses are described as spinal-ligament-derived.

Canonical marker expression supported these broad assignments. Stromal cells showed prominent expression of *COL1A1*, *DCN*, and *LUM*; Myeloid populations expressed *LST1*, *TYROBP*, and *FCER1G*; Neutrophil populations were characterized by *FCGR3B*, *S100A8*, and *S100A9*; proliferating neutrophils expressed *MKI67* and *TOP2A*; and lymphoid and vascular populations showed the expected lineage-associated marker patterns (Figure 1B).

The reconstructed resource captured the major cellular compartments across the nine-donor spinal-ligament dataset [6] and provided the transcriptional foundation for downstream genetic localization and degenerative-LSS-associated analyses.

### 3.2. LSS Genetic Susceptibility Preferentially Localizes to the Stromal Program

We next asked whether inherited susceptibility to lumbar spinal stenosis (LSS) preferentially localized to specific spinal-ligament-derived broad cellular programs. Eight broad cell-type transcriptional programs predefined from the reconstructed resource were evaluated using stratified LD score regression (S-LDSC) against the large-scale LSS genome-wide association dataset [3,12,17].

**Figure 2:**
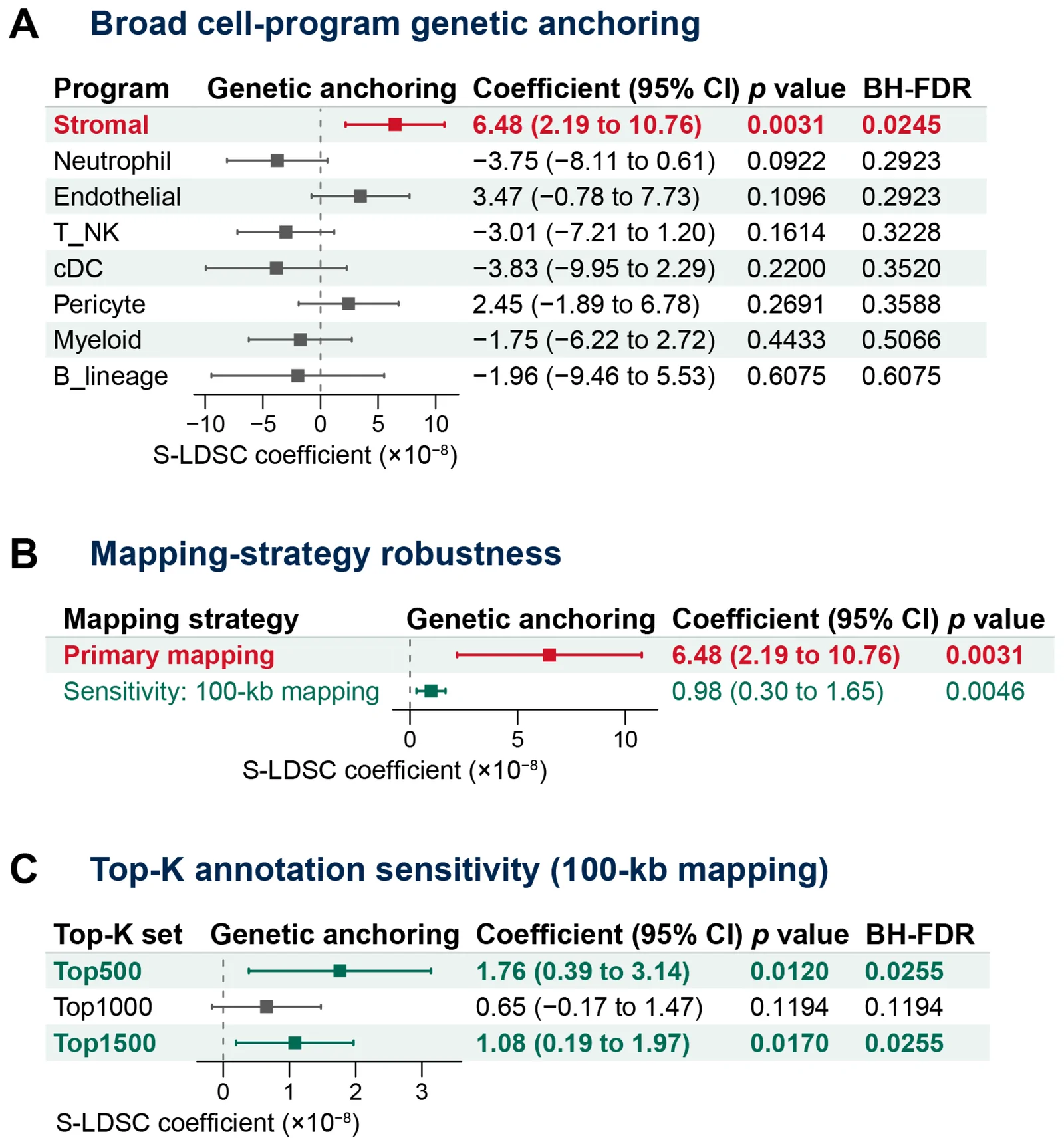
Genetic localization of spinal-ligament-derived broad cellular programs to lumbar spinal stenosis susceptibility. (**A**) S-LDSC coefficients and 95% confidence intervals for eight prespecified broad programs tested one at a time with baselineLD v2.2. (**B**) Mapping-strategy robustness comparing the primary ABC_Road_ALL broad regulatory scaffold with alternative 100 kb mapping. (**C**) Sensitivity analysis using nested Top500, Top1000, and Top1500 Stromal annotations with 100 kb mapping. Coefficients are shown on the ×10^−8^ scale; BH-FDR denotes Benjamini–Hochberg false-discovery rate. Correlation and conditional-program analyses are provided in Supplementary Figure S1. Red highlights the Stromal result in (**A**) and the primary mapping result in (**B**). Green indicates the alternative mapping result in (**B**) and the FDR-significant Top-K results in (**C**), whereas gray denotes the remaining estimates in (**A**,**C**). Alternating pale-green row shading is used solely to improve readability.

Among the eight programs, Stromal showed the strongest positive genetic anchoring signal, with an S-LDSC coefficient of 6.48 × 10^−8^ (95% CI, 2.19 × 10^−8^ to 10.76 × 10^−8^; *p* = 0.0031). The association remained significant after correction for the eight primary tests (BH-FDR = 0.0245), whereas none of the other broad cellular programs reached multiple-testing-corrected significance (Figure 2A).

The Stromal signal was robust to alternative annotation strategies. Using a conventional 100 kb gene-mapping window, the coefficient remained positive (0.98 × 10^−8^; 95% CI, 0.30 × 10^−8^ to 1.65 × 10^−8^; *p* = 0.0046) (Figure 2B). Across nested 100 kb Top-K annotations, the Top500 and Top1500 sets remained significant after correction (Top500: coefficient 1.76 × 10^−8^, 95% CI 0.39 × 10^−8^ to 3.14 × 10^−8^, BH-FDR = 0.0255; Top1500: coefficient 1.08 × 10^−8^, 95% CI 0.19 × 10^−8^ to 1.97 × 10^−8^, BH-FDR = 0.0255), whereas Top1000 did not reach significance (Figure 2C).

Correlation and conditional analyses further qualified this prioritization. Across 1,190,321 common SNPs, the Broad Stromal LD-score predictor correlated most strongly with Pericyte (Pearson r = 0.822) and Endothelial (r = 0.794); the standardized eight-predictor matrix had a condition number of 5.69. In seven pairwise conditional S-LDSC models, the Stromal coefficient remained positive and nominally significant in every model (*p* = 0.00297–0.0397), including adjustment for Pericyte (*p* = 0.0397) and Endothelial (*p* = 0.0323). Covariance-aware coefficient-difference tests remained significant after BH correction for Stromal versus Myeloid, Neutrophil, T/NK, and cDC, but not versus B lineage, Endothelial, or Pericyte. In an exploratory model containing all eight broad programs simultaneously, the Stromal coefficient remained positive (9.14 × 10^−8^) but with increased uncertainty (SE = 4.94 × 10^−8^; *p* = 0.064). Accordingly, the Broad Stromal annotation is best described as the strongest and only multiple-testing-significant positive annotation in the primary prespecified analysis, with positive pairwise conditional robustness, rather than as completely independent of correlated annotations or statistically stronger than every program [12,17] (Supplementary Figure S1).

### 3.3. Degenerative-LSS-Associated Stromal Transcription Relative to Traumatic Controls

Having genetically prioritized the Broad Stromal cellular/regulatory context, we separately examined degenerative-LSS-associated transcription within Stromal cells using donor-aware pseudobulk analysis, with donors rather than individual cells treated as biological replicates [13].

**Figure 3:**
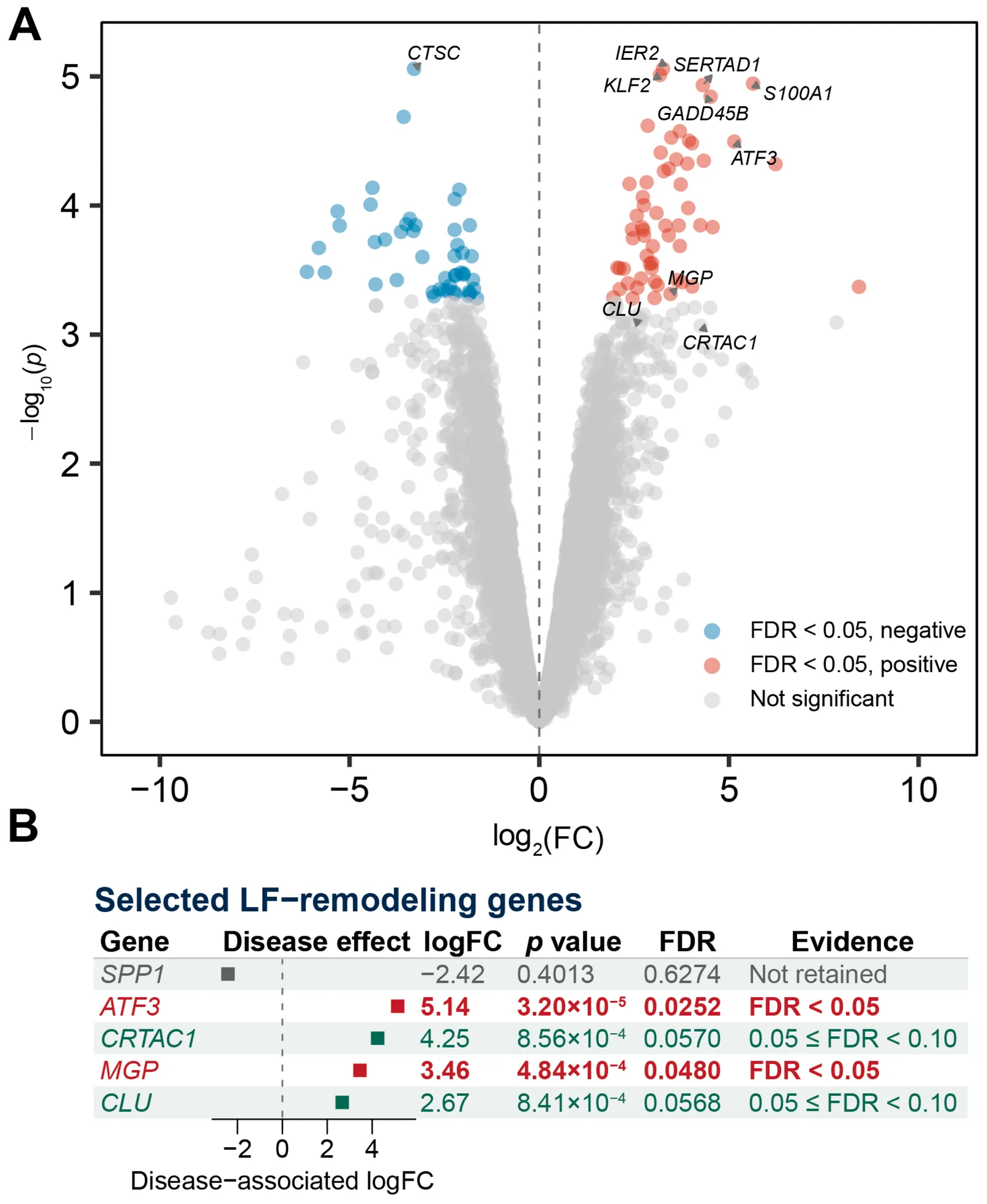
Degenerative-LSS-associated transcriptional remodeling within the genetically prioritized Stromal compartment. (**A**) Volcano plot of donor-aware Stromal pseudobulk differential expression for degenerative LSS versus traumatic control. (**B**) Degenerative-LSS-associated expression of selected LF-remodeling genes relative to traumatic controls. FDR categories reflect Benjamini–Hochberg correction across the genome-wide tested Stromal genes. In panel (**A**), blue and red points represent genes with significant negative and positive changes, respectively (FDR < 0.05), whereas gray points indicate nonsignificant genes. In panel (**B**), red indicates FDR < 0.05, green indicates 0.05 ≤ FDR < 0.10, and gray indicates genes not retained under the specified criteria.

Among 9918 evaluated autosomal genes, 106 were differentially expressed at FDR < 0.05, including 59 with higher and 47 with lower expression in degenerative-LSS relative to traumatic-control Stromal profiles (Figure 3A). The age-sensitivity audit was highly concordant with the sex-adjusted primary model across 10,774 genes (signed-Z Spearman rho = 0.989; logFC Pearson r = 0.978; 96.1% directional concordance), although adding age reduced residual degrees of freedom and substantially reduced the number of FDR-significant genes in that sensitivity analysis. These sensitivity-study FDR counts are not directly equivalent to the final 9918-gene autosomal analysis.

We then examined genes previously implicated in LF remodeling biology [4,6,7]. *ATF3* was markedly upregulated in disease-associated Stromal cells (logFC = 5.14, FDR = 0.025), and *MGP* was also significantly increased (logFC = 3.46, FDR = 0.048). *CRTAC1* (logFC = 4.25, FDR = 0.057) and *CLU* (logFC = 2.67, FDR = 0.057) showed concordant positive changes at FDR < 0.10, whereas *SPP1* did not show retained disease-associated transcriptional support within the Stromal compartment (Figure 3B).

LODO refitting indicated substantial within-cohort donor robustness. Of the 106 primary FDR-significant genes, 97 had estimates in all eight estimable LODO refits and all 97 retained the primary direction in all eight; the remaining nine genes had three to six available LODO estimates and each retained 100% directional concordance across available refits. Across the eight LODO fits, transcriptome-wide signed-Z Spearman correlations with the primary ranking ranged from 0.843 to 0.992 (median 0.968), and the primary versus gene-wise median-LODO signed-Z correlation across 9918 genes was approximately 0.999. *ATF3*, *CRTAC1*, *MGP*, *CLU*, *CRLF1*, and *MMP14* each retained 100% direction concordance across the eight estimable refits, with median LODO signed-Z values of 3.92, 3.23, 3.34, 3.13, 2.65, and −3.41, respectively [4,6]. The male-only sensitivity analysis retained the primary direction for all six genes (*ATF3* logFC = 4.73, *CRTAC1* = 3.21, *MGP* = 2.62, *CLU* = 2.01, *CRLF1* = 2.31, *MMP14* = −1.76), with genome-wide concordance rho = 0.829; because only four biological replicates were included, this result is directional sensitivity rather than independent validation [6] (Supplementary Figure S2; Supplementary Table S2).

The Broad Stromal and disease-associated Stromal analyses provided complementary but non-equivalent information [12,13]. The Broad Stromal program captured lineage-level cellular identity and showed significant primary genetic localization, whereas the disease-associated Stromal program characterized within-Stromal transcriptional differences between degenerative LSS and traumatic controls. The latter did not show significant conditioned S-LDSC support under either ABC_Road_ALL mapping (*p* = 0.774) or 100 kb mapping (*p* = 0.218) (Supplementary Figure S3). These results therefore do not establish that the observed disease-associated Stromal transcriptional state mediates inherited LSS susceptibility.

### 3.4. Cross-Layer Integration Prioritizes Genetically Supported Stromal-Oriented Loci

**Figure 4:**
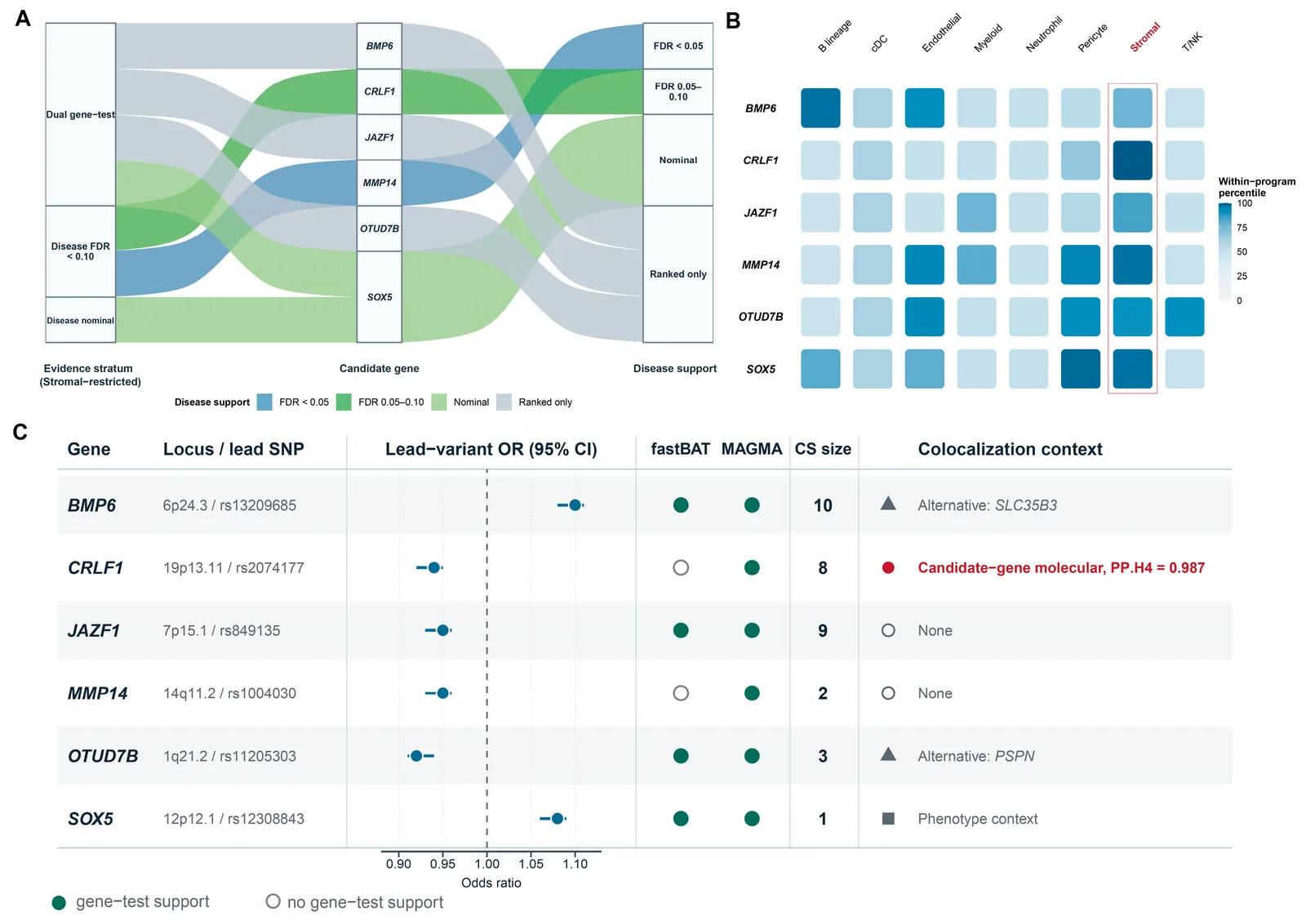
Cross-layer prioritization of genetically supported Stromal-oriented LSS loci. (**A**) Evidence-stratum flow linking transparent shortlist entry criteria to candidate genes and disease-transcription support. (**B**) Within-program percentiles of the six shortlisted genes across eight broad cellular programs; the Stromal column is highlighted. (**C**) Gene-level genetic evidence showing lead-variant odds ratios, fastBAT and MAGMA support, fine-mapping credible-set size, and target-aware exact-rsID colocalization context. Percentiles indicate within-program rank and highlight the relative Stromal orientation of prioritized candidates. (**A**) Blue, green, pale-green, and gray flows mark FDR < 0.05, 0.05 ≤ FDR < 0.10, nominal, and ranking-only support, respectively. (**B**) Darker blue indicates higher percentiles; coral outlines the Stromal column. (**C**) Filled/open green circles indicate gene-test support/absence; red dots indicate candidate-specific molecular colocalization, and gray shapes indicate alternative-target or phenotype context. Pale-gray rows aid readability.

We next integrated evidence-stratum membership, broad-program localization, disease-associated transcription, gene-level genetic association, fine-mapping, and colocalization context [3,12]. Six genes were retained for detailed cross-layer evaluation: *BMP6*, *CRLF1*, *JAZF1*, *MMP14*, *OTUD7B*, and *SOX5*, representing complementary routes into the frozen evidence set (Figure 4A).

On within-program percentile scaling, *CRLF1* ranked at the 100th percentile within the Stromal program. *MMP14* and *SOX5* each ranked at approximately the 97th percentile, with *OTUD7B* and *JAZF1* also showing high Stromal orientation and *BMP6* showing a more moderate Stromal signal (Figure 4B). These within-program rankings highlighted *CRLF1*, *MMP14*, and *SOX5* as the most strongly Stromal-oriented candidates.

Gene-level genetic evidence further differentiated the candidates. *BMP6*, *JAZF1*, *OTUD7B*, and *SOX5* were supported by both fastBAT and MAGMA, whereas *CRLF1* and *MMP14* showed MAGMA but not fastBAT support. Fine-mapping reduced the corresponding loci to credible sets ranging from a singleton signal for *SOX5* to 10 variants for *BMP6*; the *CRLF1* and *MMP14* loci contained eight and two variants, respectively (Figure 4C) [3].

Colocalization context was heterogeneous across loci [3,14]. *CRLF1* uniquely showed candidate-gene-specific molecular colocalization, with a maximum PP.H4 of 0.987 across evaluated molecular-QTL resources. In contrast, molecular colocalization at the *BMP6* and *OTUD7B* loci implicated alternative targets, *SLC35B3* and *PSPN*, respectively. *SOX5* was supported by phenotype-GWAS colocalization context rather than candidate-specific molecular-QTL evidence, whereas no exact-rsID colocalization was identified for *JAZF1* or *MMP14* (Figure 4C).

When the evidence layers were considered jointly, *CRLF1* and *MMP14* showed the most coherent overall convergence. *CRLF1* combined maximal Stromal orientation, positive disease-associated transcription (logFC = 3.49, FDR = 0.095), MAGMA support, an eight-variant credible set, and candidate-gene-specific molecular colocalization. *MMP14* combined strong Stromal orientation, significant disease-associated downregulation (logFC = −2.23, FDR = 0.033), MAGMA support, and a narrow two-variant credible set, defining a complementary extracellular-matrix remodeling candidate.

*BMP6* and *SOX5* were retained as supporting candidates on the strength of gene-level genetic evidence, while *JAZF1* and *OTUD7B* broadened the extended six-gene evidence set through complementary support. This framework yielded a structured set of loci and candidate genes for mechanistic interpretation and functional follow-up [18] (Supplementary Figure S4).

### 3.5. Regional Colocalization at the CRLF1 Locus Supports a Shared Association Signal

Because *CRLF1* showed the most complete cross-layer convergence, we examined the locus at regional resolution using the LSS GWAS and GTEx skeletal-muscle *CRLF1* cis-eQTL data [3,14].

**Figure 5:**
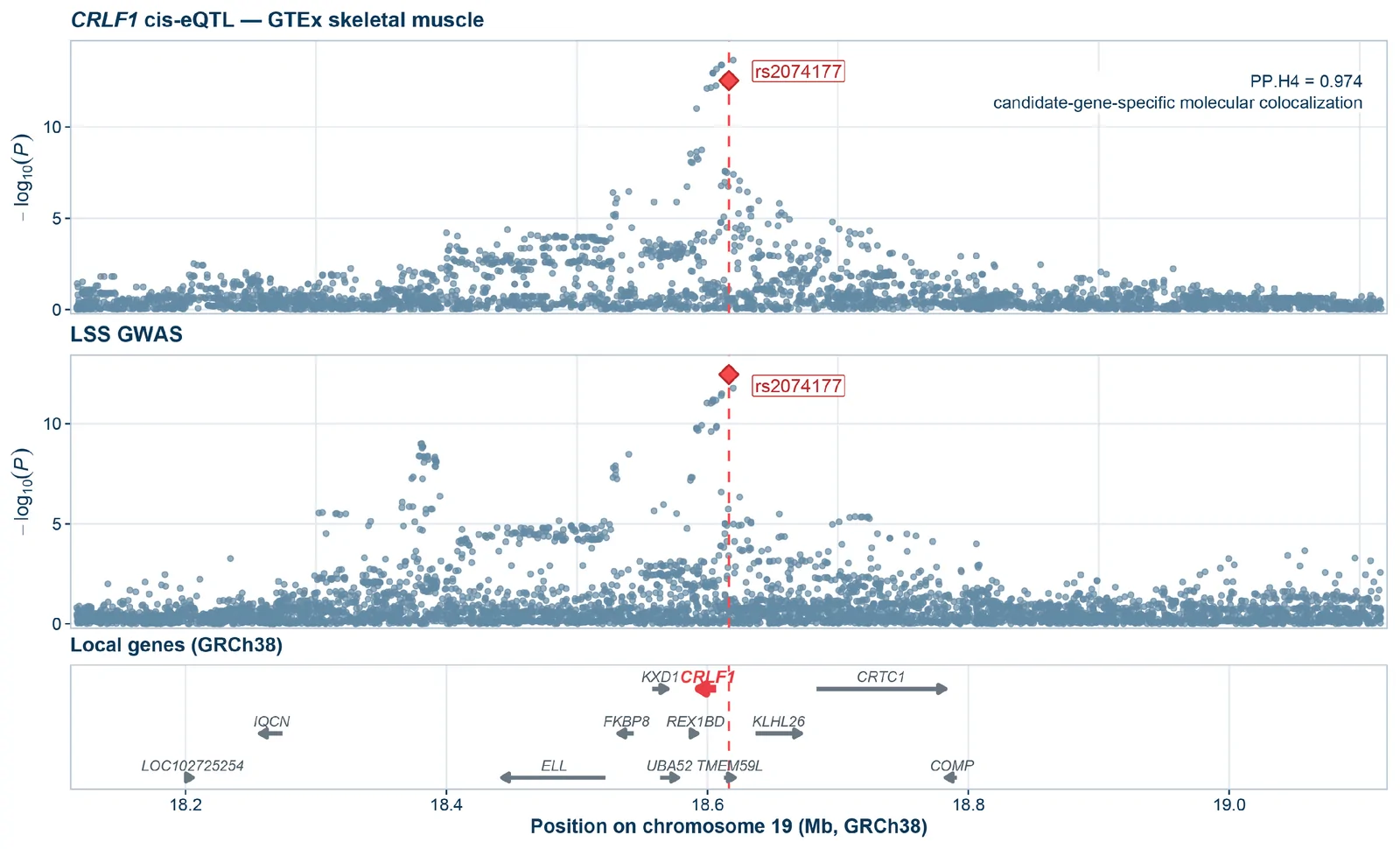
Regional molecular colocalization at the *CRLF1* locus. Regional association plots show GTEx skeletal-muscle *CRLF1* cis-eQTL signals (**top**), LSS GWAS signals (**middle**), and local genes in GRCh38 coordinates (**bottom**) across the rs2074177 region. The highlighted rs2074177 position is indicated by a red diamond and vertical dashed line. The displayed PP.H4 = 0.974 is posterior support for a shared LSS-*CRLF1* association signal in the GTEx skeletal-muscle gene-expression resource; it is not a causal probability for rs2074177. Allele harmonization indicates that the LSS risk-increasing A allele corresponds to lower *CRLF1* expression in skeletal muscle. This is cross-tissue regulatory evidence and does not establish an LF-specific mechanism. Blue-gray dots indicate variant-level association significance without LD-based color coding. Arrows in the bottom gene track indicate gene orientation, and the target gene is labeled in red. The red gene arrow identifies CRLF1, whereas gray arrows indicate neighboring genes.

Regional association data spanning approximately ±500 kb around rs2074177 on chromosome 19 included 4817 LSS GWAS variants and 3314 *CRLF1* cis-eQTL variants. The highlighted variant rs2074177 was located at chr19:18,616,479 (GRCh38), and both datasets showed prominent regional association signals centered near this locus (Figure 5).

Candidate-gene-specific colocalization between the LSS locus and the GTEx skeletal-muscle *CRLF1* cis-eQTL supported compatibility with a shared association signal (PP.H4 = 0.974) [3,14]. PP.H4 is the posterior probability assigned to the shared-signal hypothesis under the colocalization model; it is not the causal probability of rs2074177. Across the target-aware audit, *CRLF1*-specific molecular-QTL support was observed in seven resources, with a maximum PP.H4 of 0.987, while exact-rsID molecular rows at the same locus also implicated alternative targets including *REX1BD*, *KXD1*, and *TMEM59L*. Accordingly, the evidence supports *CRLF1* as a leading regulatory candidate but does not establish it as the uniquely causal gene (Figure 5 and Figure S5).

Allele harmonization further clarified direction. At rs2074177, the LSS risk-increasing allele is A and the protective allele is G; in the GTEx skeletal-muscle *CRLF1* eQTL, G is associated with higher *CRLF1* expression, so the risk-increasing A allele corresponds to lower *CRLF1* expression (equivalent beta for A versus G = −0.397042). This cross-tissue germline regulatory direction differs from the positive *CRLF1* transcription observed in degenerative-LSS-associated Stromal profiles and therefore argues against a simple risk-allele -> higher *CRLF1* -> disease mediation model. Because the eQTL source is skeletal muscle rather than ligamentum flavum, the direction cannot be assumed to represent LF regulation. Together, these findings position *CRLF1* as a leading regulatory candidate at the rs2074177 locus requiring tissue-specific functional validation [18].

### 3.6. External LF Tissue Provides Directional Sensitivity Support for CRLF1

Finally, we assessed whether the direction of prioritized transcriptional signals was observable in the external LF tissue dataset GSE113212. The prespecified frozen panel comprised the six prioritized genes together with five genes from the previously examined LF remodeling program (Supplementary Figure S6).

**Figure 6:**
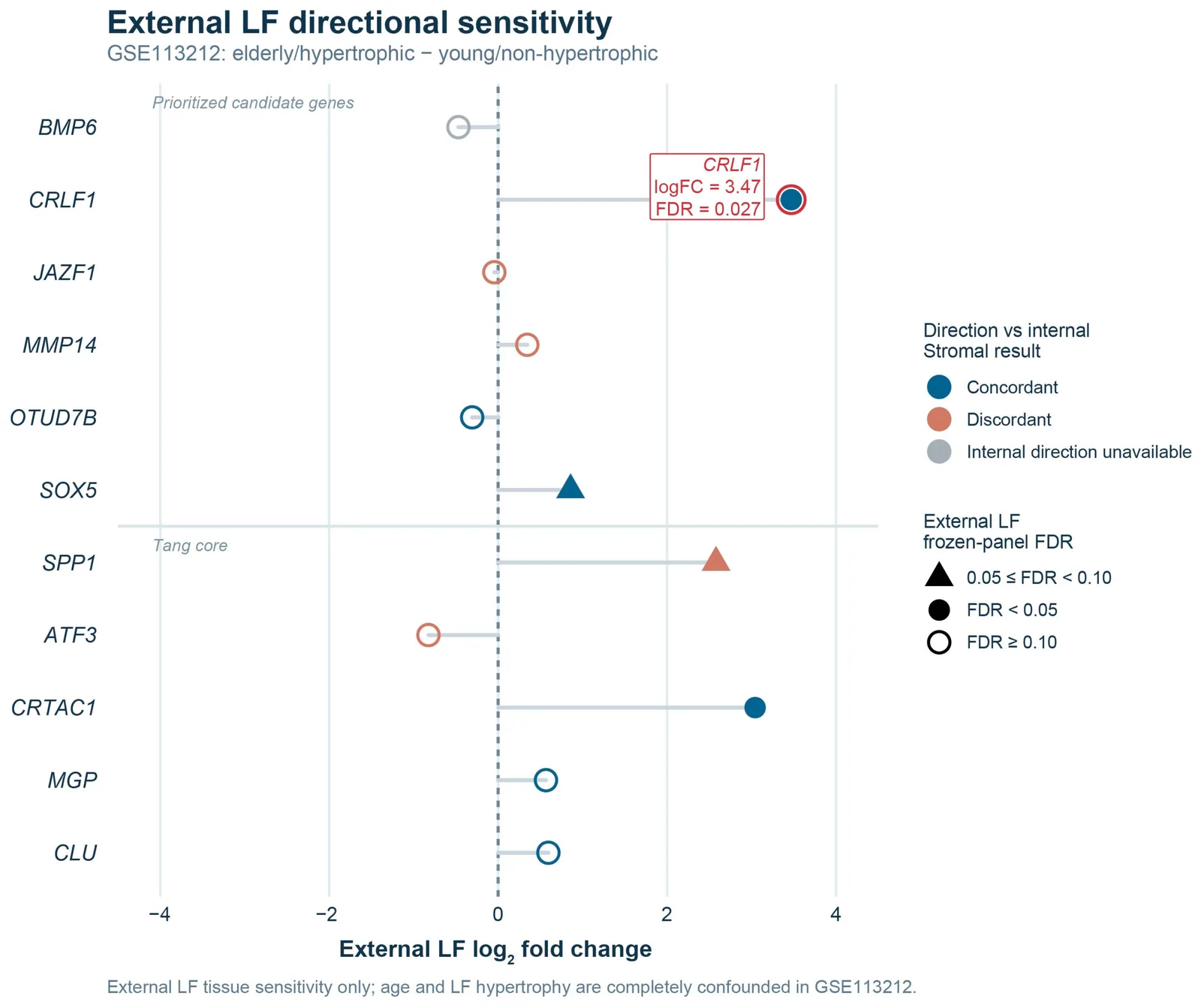
External ligamentum flavum directional-sensitivity analysis of prioritized genes. External log2 fold changes in GSE113212 are shown for the six prioritized genes and five previously examined LF-remodeling genes using the contrast elderly/hypertrophic minus young/non-hypertrophic LF. Blue symbols indicate directional concordance with the internal Stromal result, orange symbols indicate discordance, and gray symbols indicate that an internal direction was unavailable. Filled circles indicate frozen-panel FDR < 0.05, filled triangles indicate 0.05 ≤ FDR < 0.10, and open circles indicate FDR ≥ 0.10. GSE113212 contains four samples per group with age aligned to LF hypertrophy; the figure summarizes cross-dataset directional sensitivity.

*CRLF1* showed higher expression in elderly/hypertrophic LF tissue than in young/non-hypertrophic LF tissue (logFC = 3.47, *p* = 0.0049, frozen-panel BH-FDR = 0.027), concordant in direction with the internal degenerative-LSS-associated Stromal comparison (Figure 6). *SOX5* also showed a positive external signal with FDR < 0.10 (logFC = 0.86, FDR = 0.085). Because age and hypertrophy are inseparable in this dataset, the *CRLF1* difference cannot be attributed specifically to hypertrophy independent of age.

By contrast, *MMP14* and *JAZF1* were directionally discordant with their internal Stromal results. Among the previously examined LF remodeling genes, *CRTAC1* showed strong positive external expression (logFC = 3.04, FDR = 0.007), while *MGP* and *CLU* retained concordant positive directions without frozen-panel significance. *SPP1* and *ATF3* were directionally discordant relative to the internal Stromal analysis.

Among the frozen panel, *CRLF1* showed the clearest cross-dataset directional coherence; this supports its cross-layer prioritization as a sensitivity observation but does not provide age-independent or hypertrophy-specific external validation.

## 4. Discussion

### 4.1. Principal Findings

In this study, we integrated single-cell transcriptional profiling with large-scale human genetic data to localize inherited lumbar spinal stenosis (LSS) susceptibility to cellular programs defined from a spinal-ligament single-cell resource. Three findings were particularly notable. First, the Broad Stromal program was the strongest and only multiple-testing-significant positive annotation in the primary prespecified one-at-a-time S-LDSC analysis, and it remained positive and nominally significant across all seven pairwise conditional models. Second, donor-aware analysis identified a robust degenerative-LSS-associated Stromal transcriptional pattern relative to traumatic controls, but this disease-associated program itself was not significantly enriched by S-LDSC and is therefore not interpreted as a mediator of inherited susceptibility. Third, cross-layer integration of cellular localization, disease-associated transcription, gene-level association, fine-mapping, and molecular colocalization prioritized a restricted set of loci, with *CRLF1* showing the most complete candidate-specific regulatory convergence and *MMP14* providing complementary evidence through stronger disease-associated differential expression and a narrower credible set. These results distinguish lineage-level genetic localization, state-dependent transcription, and locus-level molecular evidence as separate layers of inference [3,6,12].

### 4.2. Biological and Genetic Interpretation

The preferential localization of LSS susceptibility to the Broad Stromal program provides a biologically coherent bridge between population genetics and tissue remodeling in degenerative spinal stenosis. Stromal and fibroblast-like populations are central to extracellular-matrix production, fibrotic remodeling, and chondrogenic or calcific transitions in diseased spinal ligaments [4,19,20]. Recent single-cell work identified extensive stromal heterogeneity in degenerative spinal ligaments, including fibroblast-like and chondrocyte-like populations associated with matrix degradation and calcification [6,21,22]. Our analysis extends these disease-state observations by asking a different question: whether inherited susceptibility preferentially localizes to a broad spinal-ligament Stromal cellular/regulatory context. The primary S-LDSC result supports such localization [12,17]. However, appreciable correlation with Pericyte and Endothelial annotations and the loss of nominal significance in the exploratory all-eight joint model indicate uncertainty under shared annotation structure. The pairwise conditional models support robustness of a positive Stromal signal, but the data do not establish that Stromal is statistically stronger than every other program or completely independent of correlated annotations [12,17].

The degenerative-LSS-associated Stromal analysis characterizes a separate state-dependent layer. *ATF3* and *MGP* were significantly altered, while *CRTAC1* and *CLU* showed concordant positive changes at FDR < 0.10, consistent in direction with previously described LF remodeling programs [4,6,23]. The donor-aware reanalysis emphasized Stromal remodeling centered on *ATF3*, *MGP*, *CRTAC1*, and *CLU* [6,23], but the contrast is between degenerative-LSS and traumatic-control donors rather than matched healthy controls. LODO analyses showed high within-cohort directional stability, and the male-only and age-adjusted sensitivity analyses were broadly concordant, although the small donor number limits precision and multivariable adjustment [6,13]. Critically, the disease-associated Stromal program was not significantly enriched by S-LDSC; thus, genetic localization to Broad Stromal identity and disease-associated transcription should be viewed as complementary, not as evidence that inherited variants drive or mediate the observed remodeling state [12,13].

*CRLF1* illustrates both the value and the limits of cross-layer integration. Prior experimental work already established *CRLF1* as an LF-remodeling gene, including upregulation in hypertrophic LF and involvement in TGF-β1/SMAD3- and ERK-related profibrotic pathways [9]. The novelty of the present study is therefore not rediscovery of *CRLF1* biology, but its genetic and cellular contextualization within an LSS susceptibility framework. The large LSS GWAS expanded the relevant locus map [3], and *CRLF1* emerged here through maximal Broad Stromal orientation, MAGMA support, an eight-variant credible set, and candidate-gene-specific molecular-QTL colocalization. The GTEx skeletal-muscle *CRLF1* eQTL and LSS locus were compatible with a shared signal (PP.H4 = 0.974), but PP.H4 does not identify rs2074177 as a proven causal variant or *CRLF1* as a proven causal gene. Moreover, allele harmonization showed that the LSS risk-increasing A allele corresponds to lower *CRLF1* expression in skeletal muscle, whereas *CRLF1* expression was higher in the degenerative-LSS-associated Stromal comparison. This directional difference argues against a simple linear mediation model and reinforces the need for LF-specific regulatory validation [18,24].

The remaining prioritized genes further demonstrate the value of multi-layer evidence integration [18,25]. *MMP14* showed strong Stromal orientation, significant disease-associated downregulation, MAGMA support, and a narrow two-variant credible set, nominating it as a complementary extracellular-matrix remodeling candidate [4,26]. *BMP6* and *SOX5* contributed strong gene-level genetic evidence, with distinct downstream support: the *BMP6* locus showed colocalization with an alternative molecular target, whereas *SOX5* was supported by phenotype-GWAS context rather than candidate-specific molecular-QTL evidence. *JAZF1* and *OTUD7B* broadened the shortlist through complementary combinations of evidence. These complementary evidence patterns support structured, gene-specific prioritization [18]. Each candidate is therefore positioned by its specific combination of localization, transcriptional, association, fine-mapping, and molecular-QTL evidence.

The external LF tissue analysis provided an additional but limited directional-sensitivity layer [27]. *CRLF1* was higher in elderly/hypertrophic than young/non-hypertrophic LF in GSE113212, consistent in direction with the internal Stromal result, and several remodeling genes also showed concordant directions. However, age and hypertrophy status are completely aligned in this eight-sample dataset, so age matching is infeasible and an age-adjusted model cannot resolve the design-level confounding. The result therefore demonstrates that the *CRLF1* direction is observable in a second LF expression resource, but it cannot be attributed specifically to hypertrophy or treated as independent validation [27].

### 4.3. Clinical Relevance

LF hypertrophy and fibrosis directly contribute to lumbar canal narrowing, yet current treatment is directed at established structural stenosis rather than genetically informed tissue mechanisms. By localizing inherited LSS susceptibility to a broad spinal-ligament Stromal context and narrowing cross-layer regulatory hypotheses to *CRLF1*, *MMP14*, and a small number of complementary loci, this study provides a tractable basis for experimental testing [28]. At this stage, the principal clinical value is biological prioritization rather than immediate therapeutic inference [18,28]. Future LF-specific regulatory assays and functional perturbation can focus on loci supported by population genetics while explicitly testing whether the cross-tissue regulatory observations generalize to LF stromal systems [29,30].

### 4.4. Limitations and Future Directions

Several limitations should be considered. First, GSE271018 is most conservatively described as a spinal-ligament/ligament-tissue resource; donor-level material available to us does not establish exclusive LF origin for every library. The source data were also generated after CD45-positive and CD45-negative sorting, so reconstructed cell fractions reflect the experimental sampling design and were excluded from native-abundance inference [6]. Second, the disease-expression cohort was small: the source cohort contained six degenerative-LSS and three traumatic-control donors, and Stromal eligibility yielded five versus three donors. Donor-level pseudobulk analysis and LODO refitting support within-cohort robustness, but they do not establish external reproducibility. The traumatic controls are not healthy matched controls, and residual confounding may reflect trauma-related biology, lumbar-level differences, residual sex effects, age, tissue condition, and unmeasured clinical factors. The available lumbar levels showed insufficient cross-group overlap for reliable independent adjustment, and stenosis severity and detailed trauma variables were unavailable. Third, the Broad Stromal S-LDSC signal depends on transcriptional annotation definition and gene-to-genome mapping [12,17]. Pairwise conditional analyses support a positive Stromal signal, but appreciable correlation among annotations and the exploratory all-eight joint *p* = 0.064 limit claims of annotation independence. ABC_Road_ALL is a broad, non-tissue-specific regulatory scaffold that may contain links inactive in spinal ligament, while 100 kb mapping is a robustness analysis rather than tissue-specific validation. Fourth, molecular colocalization relied on available QTL resources, including skeletal-muscle eQTL data rather than LF-specific regulatory maps [14,31]. This tissue-context gap motivates LF-specific eQTL, chromatin-accessibility, and enhancer-promoter maps [31,32,33,34]. PP.H4 indicates posterior support for a shared association signal and does not prove a causal variant, causal gene, or mediation mechanism; fine-mapping and colocalization therefore require functional validation [18,35,36]. Fifth, GSE113212 is age-hypertrophy-confounded and can provide directional sensitivity only, not independent hypertrophy-specific validation. Sixth, the exact stromal substate architecture and macrophage-to-Stromal signaling reported previously showed lower reproducibility in the present donor-aware reconstruction [6,21,22,23]. Finally, functional perturbation of prioritized variants and genes was beyond the scope of this study. Future work should use larger clinically matched spinal-ligament cohorts and LF-relevant functional systems, including allele-specific enhancer assays, CRISPR-based perturbation, and tissue-specific multi-omic mapping [25,29,31].

## 5. Conclusions

This study localizes inherited LSS susceptibility to a broad spinal-ligament Stromal cellular/regulatory context and identifies a restricted set of loci with complementary cellular, transcriptional, genetic, and regulatory evidence. *CRLF1* showed the most complete candidate-specific cross-layer regulatory convergence, placing established LF-remodeling biology within a genetically prioritized context without establishing a simple causal mediation chain [3,9]. *MMP14* provides an important complementary candidate with stronger disease-associated transcriptional support and a narrower fine-mapping credible set [26]. Together, these findings narrow the gap between LSS susceptibility loci and disease-relevant ligament biology and define testable priorities for LF-specific regulatory and functional validation [18,28,29].

## Data Availability

The datasets analyzed in this study are publicly available from external repositories. The spinal-ligament/ligament-tissue single-cell resource was obtained from the Gene Expression Omnibus (GEO) under accession GSE271018. External ligamentum flavum bulk expression data were obtained from GEO under accession GSE113212. Lumbar spinal stenosis genome-wide association summary statistics and locus-level genetic evidence were obtained from the public data release accompanying Salo et al. Variant-level *CRLF1* cis-eQTL data were obtained from the eQTL Catalogue (study QTS000015; dataset QTD000281). All derived tables underlying the revised analyses are included in the article or Supplementary Information. Analysis code and derived reproducibility materials are available as described in the Code Availability statement.

## Supplementary Materials

The following supporting information can be downloaded at: https://www.mdpi.com/article/10.3390/genes17101281/s1, Figure S1, correlation and conditional robustness of spinal-ligament-derived broad cellular annotations; Figure S2, robustness of degenerative-LSS-associated Stromal transcription relative to traumatic controls; Figure S3, disease-program S-LDSC sensitivity analyses; Figure S4, cross-layer evidence profile of six prioritized LSS loci; Figure S5, target-aware molecular colocalization audit at the *CRLF1* locus; Figure S6, GSE113212 frozen-gene expression heatmap; Table S1, donor-level metadata and uniform re-QC counts for GSE271018; Table S2, donor-robustness and covariate-sensitivity summaries for degenerative-LSS-associated Stromal transcription.

## Code Availability

The analysis code and derived reproducibility materials supporting this study are available in the versioned GitHub repository https://github.com/csh-art/LSS-stromal-genetics (accessed on 11 October 2026) and are archived on Zenodo (version v1.0.0-revision; https://doi.org/10.5281/zenodo.23213035 (accessed on 11 October 2026)). Public source datasets are available from the repositories and resources described in the Data Availability Statement.

## Abbreviations

LSS, lumbar spinal stenosis; LF, ligamentum flavum; GWAS, genome-wide association study; S-LDSC, stratified LD score regression; FDR, false-discovery rate; BH, Benjamini–Hochberg; eQTL, expression quantitative trait locus; QTL, quantitative trait locus; LODO, leave-one-donor-out; cDC, conventional dendritic cell; TMM, trimmed mean of M values; UMAP, Uniform Manifold Approximation and Projection.
