# Supplementary material for: Genetic Anchoring of Stromal Cellular Programs Relevant to Ligamentum Flavum Remodeling in Lumbar Spinal Stenosis
Source: Genes (Basel). 2026 Oct 14;17(10):1281. doi: 10.3390/genes17101281 (PMC13704665; doi:10.3390/genes17101281)
Supplement: Supplementary file 1 [file genes-17-01281-s001.zip › genes-4626154-supplementary.pdf]

Supplementary Information

Genetic Anchoring of Stromal Cellular Programs Relevant to Ligamentum Flavum Remodeling in Lumbar Spinal Stenosis

Shihe Chen, Qinglin Bai, Yufei Hua, Dong Chen, Xin Jiang*

Supplementary Figures S1–S6 and Supplementary Tables S1–S2

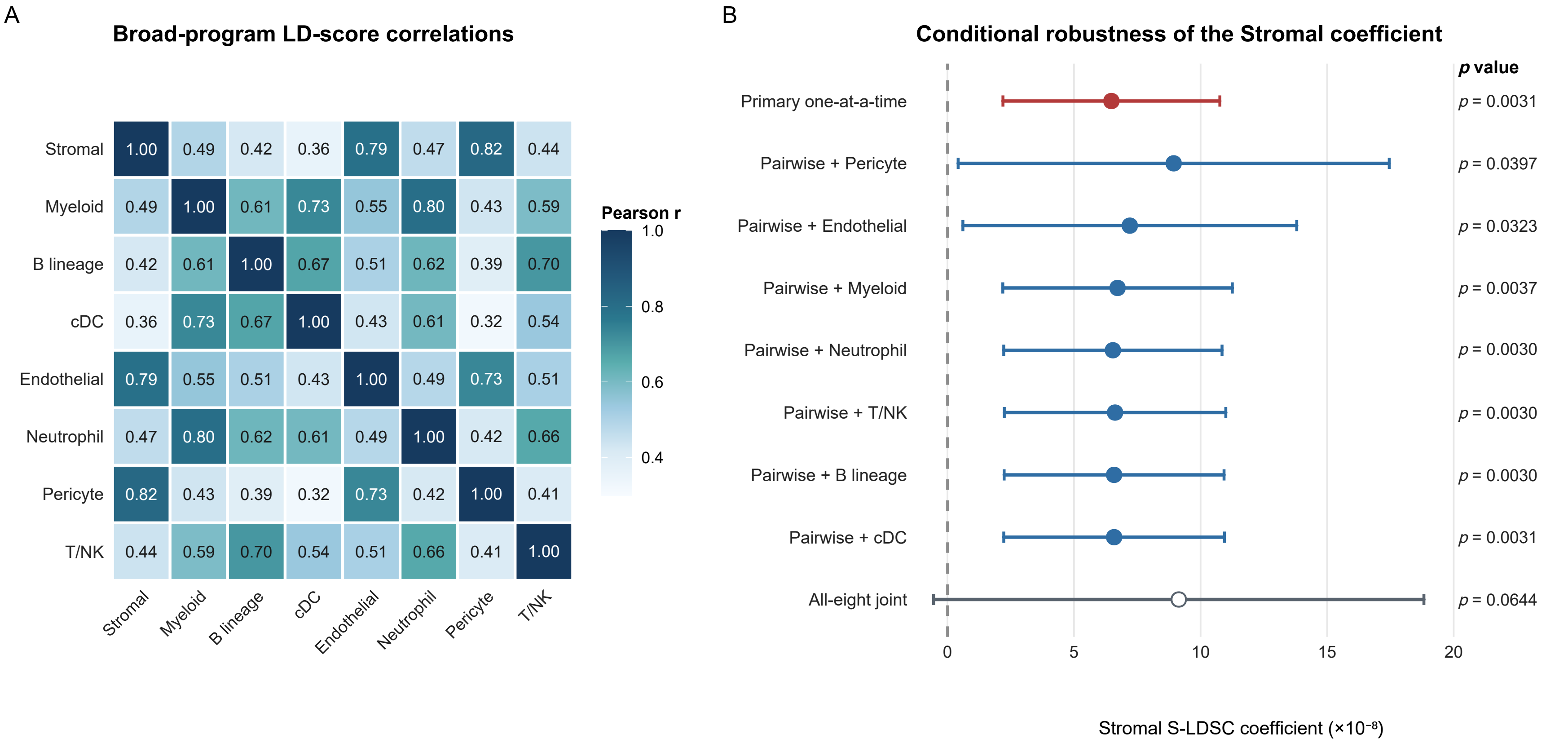

	Stromal	Myeloid	B lineage	cDC	Endothelial	Neutrophil	Pericyte	T/NK
Stromal	1.00	0.49	0.42	0.36	0.79	0.47	0.82	0.44
Myeloid	0.49	1.00	0.61	0.73	0.55	0.80	0.43	0.59
B lineage	0.42	0.61	1.00	0.67	0.51	0.62	0.39	0.70
cDC	0.36	0.73	0.67	1.00	0.43	0.61	0.32	0.54
Endothelial	0.79	0.55	0.51	0.43	1.00	0.49	0.73	0.51
Neutrophil	0.47	0.80	0.62	0.61	0.49	1.00	0.42	0.66
Pericyte	0.82	0.43	0.39	0.32	0.73	0.42	1.00	0.41
T/NK	0.44	0.59	0.70	0.54	0.51	0.66	0.41	1.00

Supplementary Figure S1. Correlation and conditional robustness of spinal-ligament-derived broad cellular annotations. (A) Pearson correlations among LD-score predictors for the eight spinal-ligament-derived broad cellular programs across 1,190,321 common SNPs. Broad Stromal showed its strongest correlations with Pericyte ($r = 0.822$) and Endothelial ($r = 0.794$). (B) Broad Stromal S-LDSC coefficients and 95% confidence intervals for the primary one-at-a-time model, seven pairwise conditional models that jointly included Broad Stromal and one companion annotation with baselineLD v2.2, and an exploratory model including all eight broad programs simultaneously. The Stromal coefficient remained positive and nominally significant in all seven pairwise models ($p = 0.00297$–0.0397). In the all-eight joint model, the coefficient remained positive (9.14×10^{-8}) but uncertainty increased and the association did not reach nominal significance ($p = 0.064$). This joint model is interpreted as an exploratory sensitivity analysis under shared annotation structure and does not indicate reversal of the primary Stromal signal.

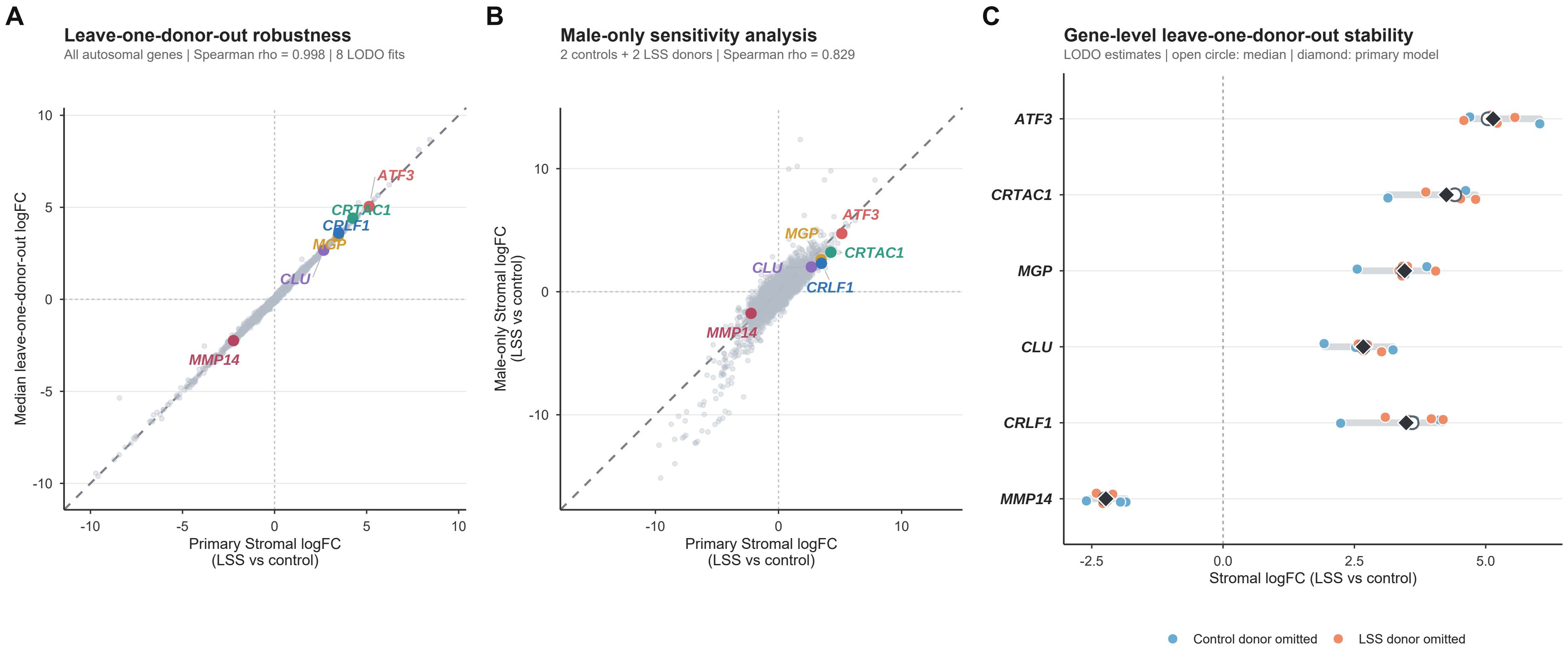

Supplementary Figure S2. Robustness of degenerative-LSS-associated Stromal transcription relative to traumatic controls. (A) Primary versus median leave-one-donor-out (LODO) Stromal logFC estimates across autosomal genes (Spearman $\rho = 0.998$). (B) Primary versus male-only estimates using two traumatic-control and two degenerative-LSS donors ($\rho = 0.829$); this four-donor analysis is interpreted only as directional sensitivity. (C) LODO estimates for *ATF3*, *CRTAC1*, *MGP*, *CLU*, *CRLF1*, and *MMP14*; open circles denote LODO medians and diamonds denote the primary-model estimates.

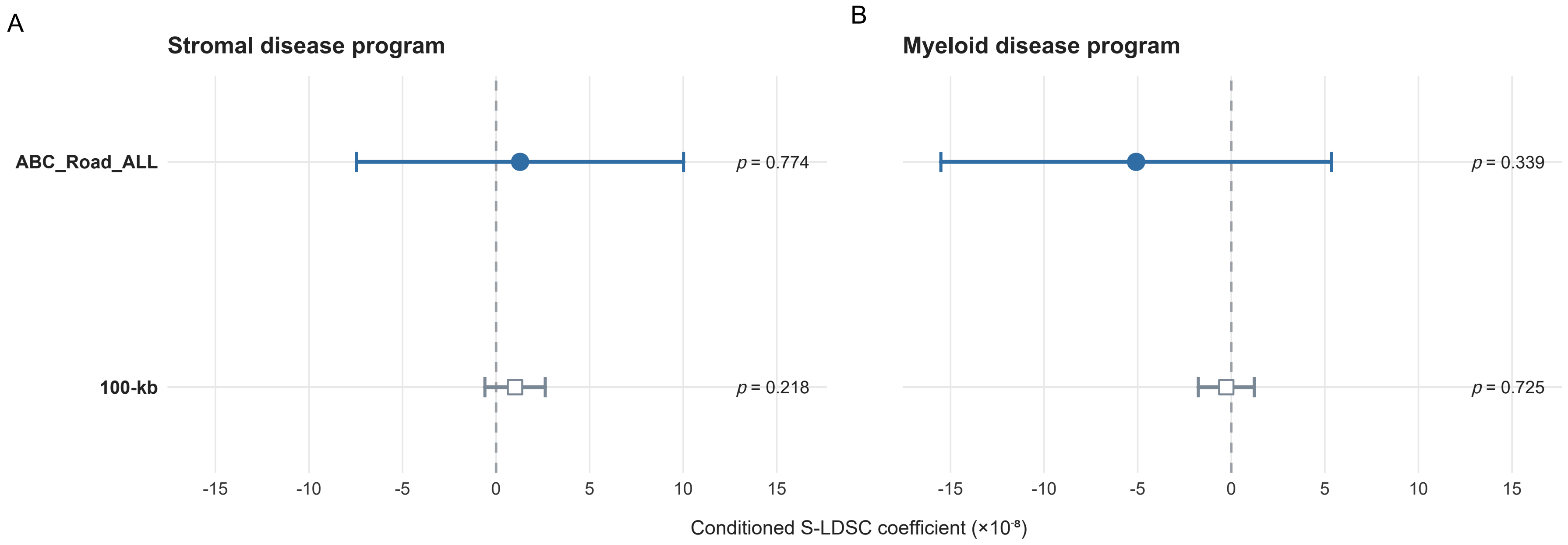

Supplementary Figure S3. Disease-program S-LDSC sensitivity analyses. Conditioned stratified LD score regression (S-LDSC) coefficients and 95% confidence intervals for the degenerative-LSS-associated Stromal (A) and Myeloid (B) programs using ABC_Road_ALL and 100-kb mapping. p values are two-sided; coefficients are shown on the $\times 10^{-8}$ scale. These disease-state programs are analytically distinct from the Broad Stromal lineage program used for primary genetic localization.

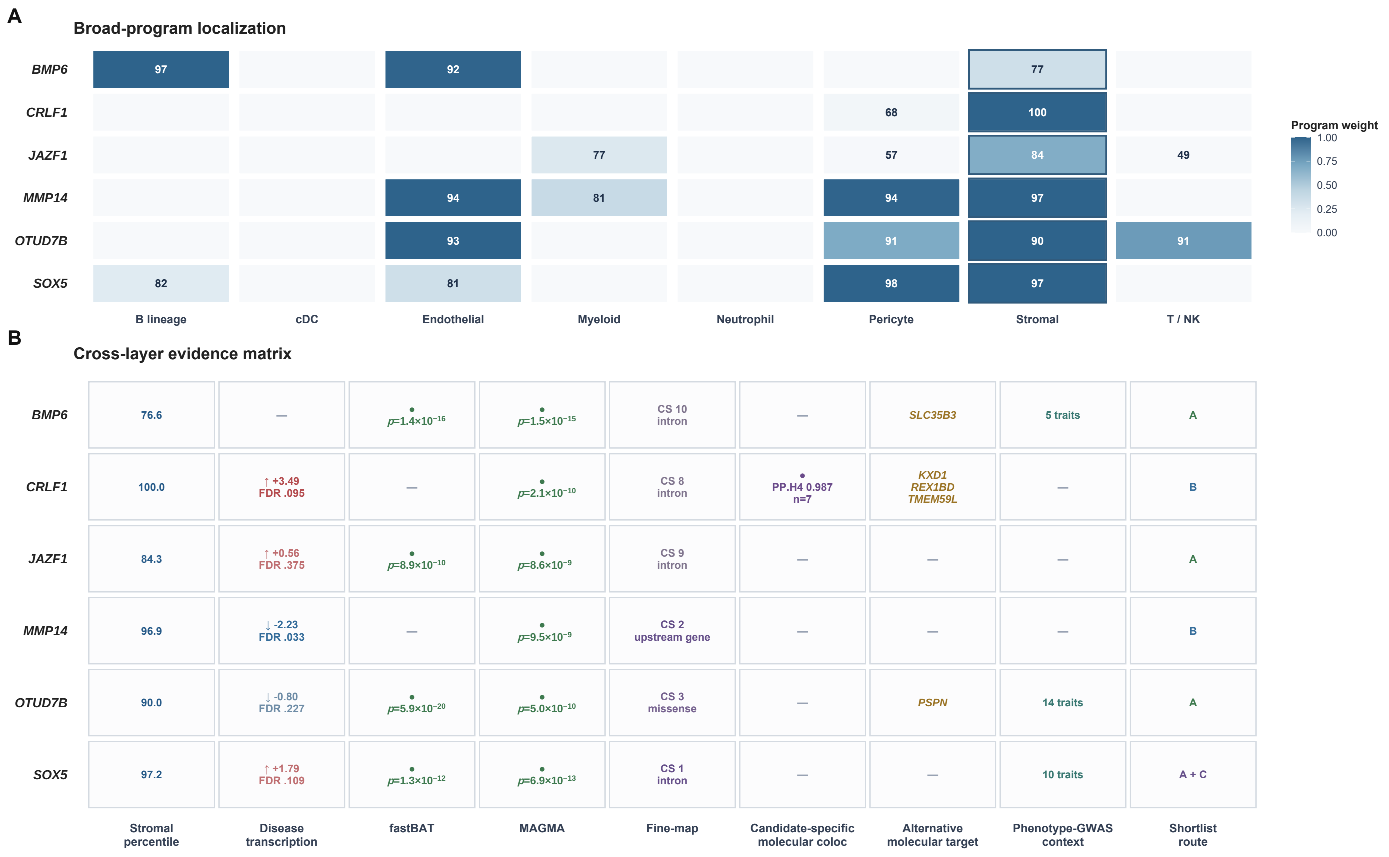

Supplementary Figure S4. Cross-layer evidence profile of six prioritized LSS loci. (A) Broad-program localization of *BMP6*, *CRLF1*, *JAZF1*, *MMP14*, *OTUD7B*, and *SOX5* across eight spinal-ligament-derived cellular programs. Fill denotes the 0–1 program weight; numbers are within-program percentiles for positive weights. (B) Evidence matrix summarizing Stromal percentile, degenerative-LSS-associated transcription relative to traumatic controls, fastBAT, MAGMA, credible-set size, candidate-specific and alternative-target molecular colocalization, phenotype-GWAS context, and shortlist route. Routes: A, fastBAT + MAGMA + positive Stromal weight; B, gene test + positive Stromal weight + disease FDR < 0.10; C, gene test + positive Stromal weight + nominal disease transcription.

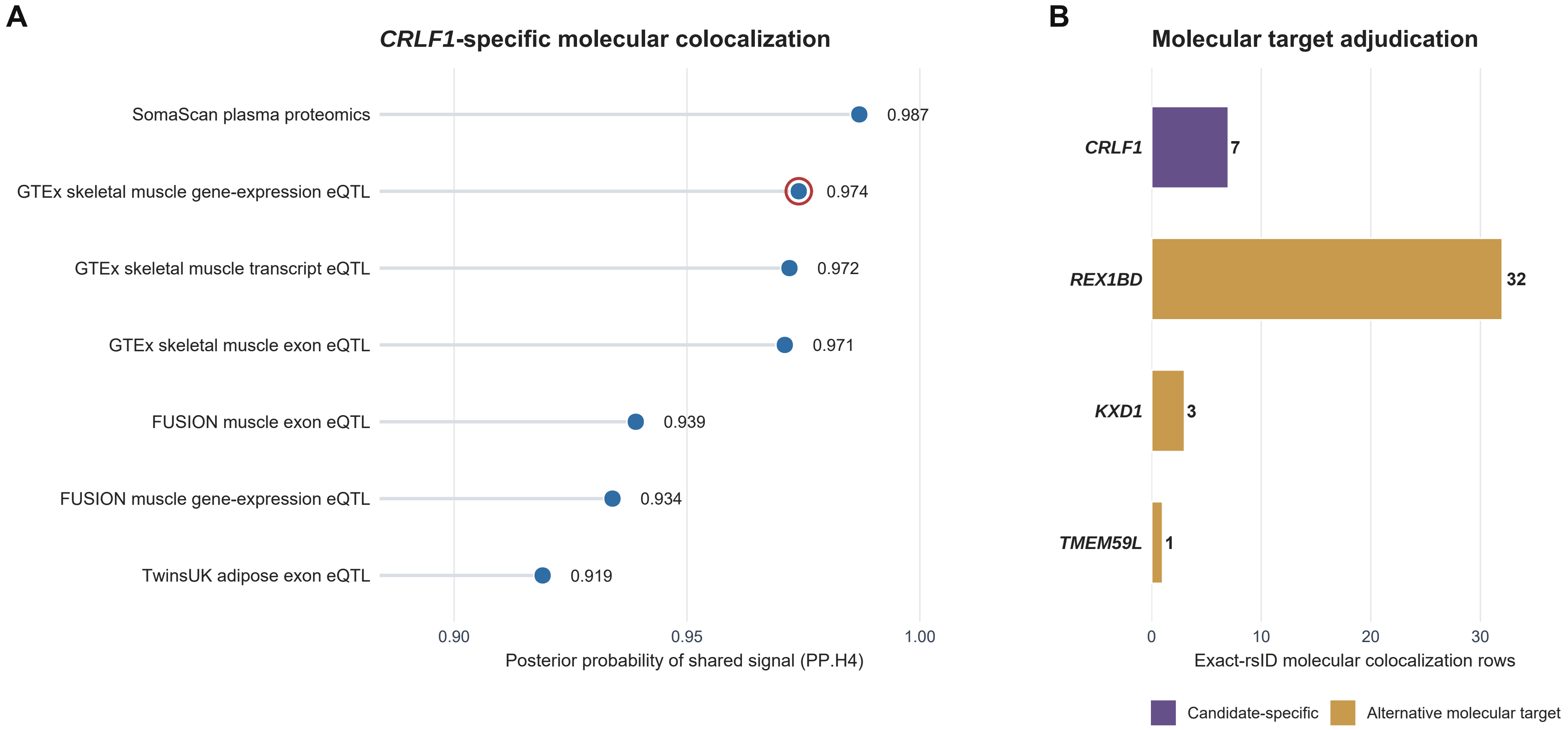

Supplementary Figure S5. Target-aware molecular colocalization audit at the *CRLF1* locus. (A) PP.H4 values for seven *CRLF1*-specific molecular-QTL colocalizations identified by exact lead-rsID matching. PP.H4 denotes posterior support for a shared association signal under the colocalization model and is not the causal probability of rs2074177. The red outline marks the GTEx skeletal-muscle gene-expression eQTL used in Figure 5. (B) Exact-rsID molecular colocalizations assigned to *CRLF1* or alternative targets at the same locus (*REX1BD*, *KXD1*, and *TMEM59L*). The molecular-QTL resources shown are not LF-specific.

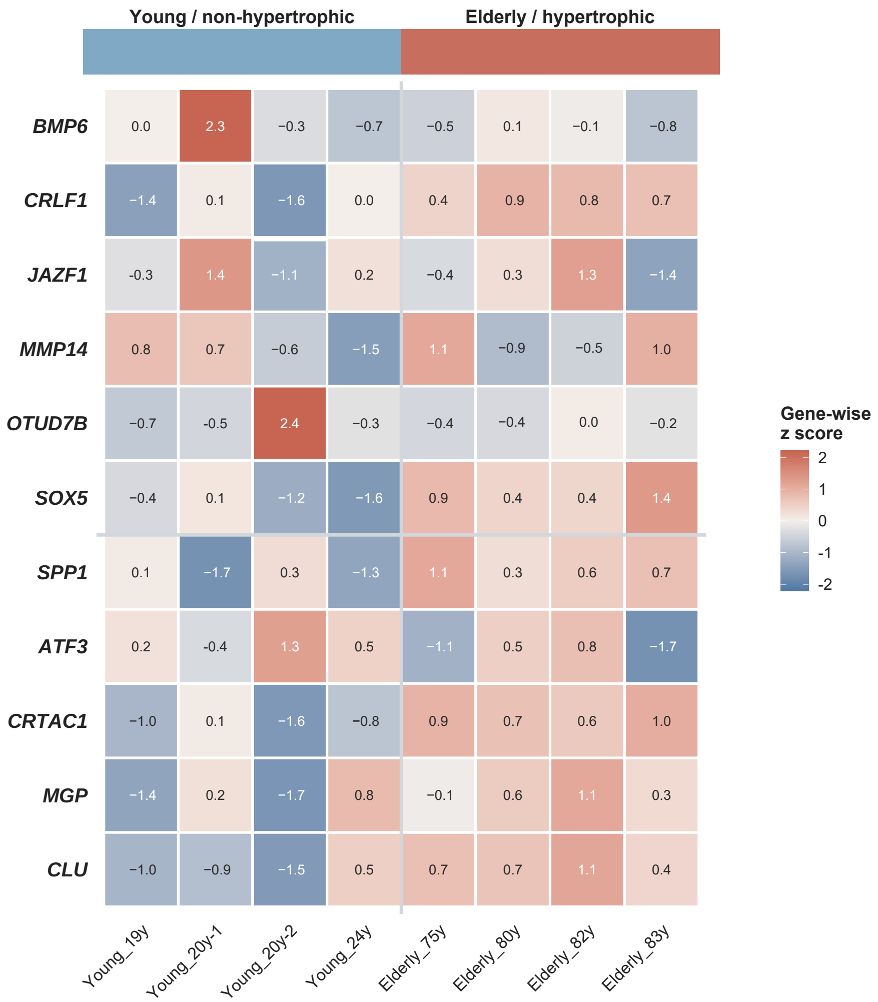

Descriptive supplementary figure only; age and LF hypertrophy are completely aligned in GSE113212.

Supplementary Figure S6. GSE113212 frozen-gene expression heatmap. Gene-wise z scores for 11 prespecified genes across four young/non-hypertrophic and four elderly/hypertrophic ligamentum flavum (LF) samples. The first six genes are prioritized candidates and the remaining five are reference LF-remodeling genes. Red and blue indicate higher and lower expression relative to each gene mean. Because age and hypertrophy status are completely aligned and the age distributions do not overlap, age matching is not feasible within GSE113212; this analysis is descriptive and is used only as external directional sensitivity.

Supplementary Table S1. Donor-level metadata and uniform re-QC counts for GSE271018.

Donor	Group	Sex	Age	Lumbar level	Cells before uniform QC	Cells retained
**Tendon_N0**	Degenerative LSS	Female	49	L5	3,337	3,337
**Tendon_N1**	Degenerative LSS	Female	51	L4	3,185	3,185
**Tendon_N2**	Degenerative LSS	Female	74	L4-L5	3,065	3,065
**Tendon_N3**	Degenerative LSS	Female	51	L3-L4	2,823	2,823
**Tendon_N5**	Degenerative LSS	Male	57	L5	5,149	5,149
**Tendon_N10**	Degenerative LSS	Male	60	L4	952	952
**Tendon_C1**	Traumatic control	Female	57	L2	7,211	3,109
**Tendon_C3**	Traumatic control	Male	51	L1	21,142	16,602
**Tendon_C5**	Traumatic control	Male	56	L3	4,822	4,822

Notes: The source study describes these libraries as spinal-ligament/ligament-tissue samples and provides lumbar sampling levels, but the donor-level material used here does not establish that every library was derived exclusively from ligamentum flavum. Live CD45-positive and CD45-negative fractions were separated before single-cell preparation; project records indicate fixed-ratio mixing. The downloaded donor-level objects contained 51,686 cells before uniform re-QC, of which 43,044 were retained.

Supplementary Table S2. Donor-robustness and covariate-sensitivity summaries for degenerative-LSS-associated Stromal transcription.

A. Genome-wide robustness and covariate sensitivity

Audit item	Result	Interpretation
Primary autosomal Stromal genes tested	9,918	Donor-aware pseudobulk analysis
Primary FDR < 0.05 genes	106 (59 higher in LSS; 47 higher in controls)	Primary DEG set
DEGs with all 8 estimable LODO refits	97/106; all 97 were 8/8 directionally concordant	No observed sign reversal
DEGs with fewer available LODO estimates	9/106; 3–6 available refits; 100% directional concordance	No observed sign reversal in available refits
Per-refit transcriptome-wide signed-Z Spearman rho	0.843–0.992; median 0.968	Broad stability with variable donor influence
Primary vs gene-wise median LODO signed-Z	rho = 0.999	Very high overall concordance
Male-only sensitivity	$n = 4$ donors (2 LSS, 2 traumatic controls); genome-wide rho = 0.829	Directional sensitivity only; not independent replication
Sex-plus-age sensitivity	10,774 common genes; signed-Z rho = 0.989; logFC $r = 0.978$; 96.1% direction concordance	Broad effect-direction stability with reduced multivariable power
Age-sensitivity FDR counts	82 to 5 after adding age in the S02B audit	Power loss after one additional covariate; not directly equivalent to the final 106-gene autosomal set

Supplementary Table S2 (continued). B. Principal-gene donor robustness

Gene	Primary logFC	Primary signed Z	Median LODO signed Z	LODO direction	Male-only logFC	Male-only direction
ATF3	5.141	4.159	3.922	100%	4.731	Concordant
CRTAC1	4.251	3.334	3.234	100%	3.207	Concordant
MGP	3.458	3.489	3.339	100%	2.615	Concordant
CLU	2.668	3.339	3.130	100%	2.012	Concordant
CRLF1	3.485	2.746	2.653	100%	2.309	Concordant
MMP14	-2.232	-3.784	-3.411	100%	-1.757	Concordant

LODO, leave-one-donor-out. Donor omission was evaluated within the source cohort and supports within-cohort robustness; it does not establish external reproducibility. The male-only analysis contains only four biological replicates and is interpreted as directional sensitivity. The age-adjusted audit was likewise treated as a sensitivity analysis because the small donor number limits multivariable precision.
